# Adjuvant disitamab vedotin plus PD-1 blockade and gemcitabine/cisplatin in high-risk upper tract urothelial carcinoma: a two-stage real-world comparative study

**DOI:** 10.3389/fimmu.2026.1809094

**Published:** 2026-06-16

**Authors:** Cheng Wang, Shuaipeng He, Dan Li, Biao Zhang, Yu Dai, Can Li, Xuan Li, Le Kang, Shujun Yang, Su Zhang, Ning Fan, Hong Chang, Gongjin Wu, Zhongjin Yue, Junhai Ma, Panfeng Shang

**Affiliations:** 1 Department of Urology, The Second Hospital of Lanzhou University, Lanzhou, Gansu, China; 2 Lanzhou University, Lanzhou, Gansu, China; 3 Department of Urology, Baoji People’s Hospital, Baoji, Shaanxi, China

**Keywords:** adjuvant therapy, antibody–drug conjugate, disitamab vedotin, HER2, PD-1 blockade, real-world evidence, upper tract urothelial carcinoma

## Abstract

**Objectives:**

To evaluate real-world effectiveness and safety of adjuvant disitamab vedotin plus PD-1 blockade (ADC+ICI) versus gemcitabine/cisplatin (GC) in high-risk upper tract urothelial carcinoma after radical nephroureterectomy.

**Patients and methods:**

This single-center, two-stage observational study enrolled 421 patients with study-defined high-risk upper tract urothelial carcinoma. Cohort A evaluated adjuvant GC versus surgery alone using propensity score matching. Cohort B compared adjuvant GC with ADC+ICI using overlap weighting, with ECOG performance status and three-level HER2 IHC category included in covariate adjustment. Overall survival (OS), conventional disease-free survival (DFS), non-intravesical progression-free survival (PFS), intravesical recurrence-free survival (IVRFS), safety, and exploratory HER2-stratified outcomes were assessed.

**Results:**

In Cohort A, adjuvant GC was associated with improved OS after matching. In Cohort B, 48 patients received GC and 53 received ADC+ICI. After overlap weighting, baseline covariates were well balanced. ADC+ICI was not associated with a significant OS improvement versus GC (HR 0.47, 95% CI 0.12–1.89; P = 0.287; FDR-adjusted P = 0.403). Directionally favorable associations were observed for conventional DFS (HR 0.32, 95% CI 0.11–0.96; nominal P = 0.043; FDR-adjusted P = 0.172), non-intravesical PFS (HR 0.32, 95% CI 0.09–1.18; P = 0.086), and IVRFS (HR 0.21, 95% CI 0.04–1.09; P = 0.063). HER2 IHC 2+/3+ tumors showed exploratory favorable DFS/PFS signals. Grade ≥3 treatment-related adverse events were similar between groups.

**Conclusions:**

Adjuvant ADC+ICI showed comparable short-term OS and directionally favorable DFS/PFS trends versus GC, with a distinct toxicity profile. These findings are hypothesis-generating and require prospective validation.

## 1 Introduction

Upper tract urothelial carcinoma (UTUC) is an uncommon but clinically aggressive malignancy, accounting for approximately 5–10% of urothelial carcinomas (1). Radical nephroureterectomy (RNU) with bladder cuff excision remains the standard treatment for localized high-risk disease. However, postoperative recurrence remains a major clinical challenge, particularly in patients with adverse pathological features such as muscle-invasive disease, lymphovascular invasion, nodal involvement, or positive surgical margins. Despite curative-intent surgery, a substantial proportion of high-risk patients experience locoregional recurrence, distant metastasis, or intravesical recurrence, resulting in unsatisfactory long-term survival outcomes. Therefore, effective and tolerable adjuvant treatment strategies remain essential for improving postoperative disease control in high-risk UTUC (2, 3).

The POUT trial established the role of platinum-based adjuvant chemotherapy by demonstrating improved disease-free survival after RNU in patients with pT2–T4 pN0–N3 M0 UTUC (4, 5). Accordingly, platinum-based adjuvant chemotherapy, most commonly gemcitabine plus cisplatin, is recommended for appropriately selected high-risk patients. Nevertheless, implementation in routine practice remains challenging. RNU removes a functional renal unit and may lead to postoperative decline in renal function, while advanced age, comorbidities, impaired performance status, and treatment-related toxicities may further limit the use or completion of cisplatin-based chemotherapy. These considerations have created a clinical need for postoperative treatment strategies that are both effective and tolerable in patients with limited suitability for standard platinum-based therapy (6, 7).

Disitamab vedotin is a HER2-targeting antibody–drug conjugate that has shown antitumor activity in advanced urothelial carcinoma, including real-world studies of locally advanced or metastatic UTUC. In parallel, PD-1 blockade has become an important component of systemic therapy for urothelial carcinoma, and combination strategies involving antibody–drug conjugates and immune checkpoint inhibitors are biologically plausible because ADC-mediated tumor cell injury may enhance antigen release and immune activation (8, 9). However, evidence supporting the use of disitamab vedotin plus PD-1 blockade in the postoperative adjuvant setting of high-risk UTUC remains limited, and comparative data against standard gemcitabine/cisplatin are lacking. Therefore, we conducted a single-centre, two-stage real-world observational study to compare adjuvant disitamab vedotin plus PD-1 blockade with gemcitabine/cisplatin after RNU. The study aimed to evaluate short-term effectiveness, safety, treatment feasibility, and exploratory HER2-stratified signals, while recognizing the non-randomized design and the hypothesis-generating nature of the analysis.

## 2 Patients and methods

### 2.1 Study design and patient selection

This was a single-centre, two-stage real-world observational study conducted at Lanzhou University Second Hospital (**Supplementary Figure 1**). The study protocol was approved by the Ethics Committee of the Second Hospital of Lanzhou University and was conducted in accordance with the Declaration of Helsinki and relevant national regulations. All eligible patients included in the present analysis were treated and followed at Lanzhou University Second Hospital. No patient-level data from Baoji People’s Hospital or any other external institution were included.

The study included two cohorts. Cohort A was a retrospective cohort of patients treated between January 2012 and December 2021 and was used to evaluate the effectiveness of adjuvant gemcitabine/cisplatin (GC) compared with surveillance after radical nephroureterectomy (RNU). Cohort B was a prospectively followed observational cohort of patients treated between January 2022 and April 2025, in which adjuvant GC was compared with disitamab vedotin plus PD-1 blockade (ADC+ICI) in contemporary routine clinical practice.

Patients were eligible if they had histologically confirmed UTUC, underwent RNU with bladder cuff excision, had no evidence of distant metastasis at baseline, and met the study-defined high-risk criteria. Study-defined high-risk disease was defined as the presence of at least one of the following features: high-grade tumor, pathological stage ≥pT2, lymphovascular invasion, pathologically or clinically suspected nodal involvement, or positive surgical margin. These criteria were intended to capture a broad real-world high-risk population rather than to exactly replicate the eligibility criteria of randomized adjuvant trials.

Patients were excluded if they had received neoadjuvant systemic therapy, had distant metastasis at diagnosis, had concurrent or previous malignancy within five years, had incomplete key baseline or follow-up data, or were considered unsuitable for the assigned systemic treatment according to routine clinical assessment.

### 2.2 Surgical intervention and pathological assessment

All patients underwent RNU with bladder cuff excision according to standard institutional practice. Surgical approach and the extent of lymph node dissection were determined by the operating surgeon based on tumor location, preoperative imaging, intraoperative findings, and patient condition. Lymph node dissection was performed when suspicious lymphadenopathy was identified on imaging or intraoperatively, or when considered clinically indicated by the surgical team.

Pathological staging was assigned according to the 8th edition of the American Joint Committee on Cancer TNM classification. Tumor grade, lymphovascular invasion, surgical margin status, tumor location, tumor multifocality, and nodal status were extracted from pathology reports and verified by the study team. HER2 expression was evaluated by immunohistochemistry when tissue was available. In the revised analysis, HER2 expression was categorized as IHC 0, IHC 1+, and IHC 2+/3+ to avoid grouping HER2-low tumors with HER2-null disease. HER2-stratified analyses were exploratory.

### 2.3 Adjuvant treatment protocols

In Cohort A, patients received either surveillance after RNU or adjuvant GC according to physician recommendation, postoperative renal function, patient preference, and treatment availability during the retrospective study period. The GC regimen generally consisted of gemcitabine plus cisplatin for 4–6 cycles, with dose adjustment according to renal function, hematological toxicity, and overall tolerance.

In Cohort B, treatment allocation was not randomized or protocol-mandated. The choice between adjuvant GC and ADC+ICI was made through multidisciplinary team discussion and shared decision-making with patients. The decision took into account postoperative renal function, ECOG performance status, pathological risk features, HER2 expression, comorbidities, anticipated platinum tolerance, drug availability, financial considerations, and patient preference.

Patients in the GC group received gemcitabine plus cisplatin according to institutional practice. Dose reductions, treatment delays, or switching from cisplatin to carboplatin were permitted when clinically indicated because of renal function decline, hematological toxicity, or intolerance. Patients in the ADC+ICI group received disitamab vedotin in combination with a PD-1 inhibitor, either toripalimab or tislelizumab, according to drug availability and physician–patient decision-making. Treatment was planned for 4–6 cycles/doses where feasible and was discontinued in cases of disease recurrence or progression, unacceptable toxicity, patient decision, financial reasons, transfer of care, or other non-medical reasons. Postoperative platinum suitability was assessed using available clinical information. In the revised analysis, partial cisplatin-ineligibility was defined as eGFR <60 ml/min/1.73 m² or ECOG performance status ≥2. Full Galsky-defined cisplatin eligibility could not be reconstructed because baseline hearing impairment, pre-existing peripheral neuropathy grade, and NYHA cardiac function were not uniformly recorded (10).

Because ADC+ICI was not an established standard adjuvant regimen for UTUC during the study period, it was administered as individualized off-label postoperative therapy after MDT discussion and clinical informed decision-making. The present study analyzed de-identified observational data and did not assign treatment by protocol. Because treatment was determined in routine clinical practice rather than assigned by an interventional study protocol, this observational analysis was not registered as a clinical trial.

### 2.4 Survival endpoints and follow-up

Follow-up was performed according to institutional practice and included clinical assessment, laboratory testing, cystoscopy, urinary tract imaging, and chest/abdominal imaging as clinically indicated. In general, patients were followed every three months during the first two years after RNU and every six months thereafter. Follow-up information was obtained from outpatient visits, inpatient records, imaging reports, cystoscopy reports, pathology records, and telephone follow-up when necessary.

Overall survival (OS) was defined as the time from RNU to death from any cause. Cancer-specific survival (CSS) was defined as the time from RNU to death attributed to UTUC. Conventional disease-free survival (DFS) was defined as the time from RNU to any recurrence, progression, or death, including intravesical recurrence, locoregional recurrence, nodal recurrence, distant metastasis, or death from any cause, whichever occurred first.

The originally defined progression-free survival endpoint was clarified in the revised manuscript as non-intravesical progression-free survival. Non-intravesical PFS was defined as the time from RNU to locoregional recurrence, nodal recurrence, or distant metastasis, whichever occurred first. Intravesical recurrence was not counted as a non-intravesical PFS event. Intravesical recurrence-free survival (IVRFS) was defined as the time from RNU to first cystoscopically and/or histologically confirmed bladder recurrence. Metastasis-free survival (MFS) was defined as the time from RNU to first distant metastasis.

Adverse events were graded according to the Common Terminology Criteria for Adverse Events, version 5.0. Treatment-related adverse events were extracted from medical records and treatment follow-up documentation.

### 2.5 Statistical analysis

Continuous variables are presented as median and interquartile range, and categorical variables are presented as number and percentage. Between-group comparisons were performed using the Wilcoxon rank-sum test, chi-square test, or Fisher’s exact test, as appropriate. All statistical tests were two-sided, and nominal P values <0.05 were considered statistically significant unless otherwise specified.

In Cohort A, propensity score matching was used to compare patients receiving adjuvant GC with those managed by surveillance after RNU. Propensity scores were estimated using logistic regression incorporating prespecified baseline covariates, including demographic characteristics, comorbidities, tumor characteristics, perioperative factors, and laboratory indices. Patients were matched using nearest-neighbor matching at a 1:2 ratio with a caliper of 0.1 on the logit of the propensity score.

In Cohort B, overlap weighting was used to compare adjuvant GC with ADC+ICI (11). The propensity score model included age, sex, body mass index, symptoms, diabetes, hypertension, hydronephrosis, nodal status, T stage, tumor location, tumor size, tumor side, tumor grade, surgical margin, lymphovascular invasion, history of bladder cancer, ureteroscopic examination, postoperative intravesical instillation, hemoglobin, eGFR, systemic immune–inflammation index, surgery year, ECOG performance status, and HER2 IHC category. Partial cisplatin-ineligibility based on eGFR/ECOG was additionally assessed and reported in the baseline and covariate-balance assessments.

Survival outcomes were estimated using the Kaplan–Meier method and compared using Cox proportional hazards models in matched or weighted cohorts. For IVRFS, death before intravesical recurrence was considered a competing event, and Fine–Gray subdistribution hazard models were used as sensitivity analyses. Restricted mean survival time was calculated for selected endpoints when clinically appropriate.

Because multiple endpoints and exploratory subgroup analyses were evaluated, Benjamini–Hochberg false discovery rate adjustment was applied to the main Cohort B survival models and exploratory HER2-stratified analyses. Both nominal and FDR-adjusted P values are reported where appropriate.

Prespecified and exploratory sensitivity analyses included conventional DFS analysis, non-intravesical PFS analysis, PD-1 inhibitor type analysis within the ADC+ICI group, HER2 IHC 0/1+/2+/3+ stratified analyses, exclusion of GC patients who switched from cisplatin to carboplatin, and competing-risk analyses for IVRFS. Formal treatment-by-HER2 interaction testing was attempted but was interpreted cautiously because of sparse event counts within HER2 strata.

All analyses were performed using R software. Survival analyses were conducted using standard survival-analysis packages, and propensity score balance was assessed using standardized mean differences and graphical diagnostics.

### 2.6 Ethics and informed consent

The ADC+ICI regimen was not assigned by the study protocol. It was administered as individualized off-label adjuvant therapy after MDT discussion and clinical informed decision-making in routine practice. The ethics committee approved the observational analysis of de-identified clinical data and waived the requirement for additional written informed consent for the present study. The waiver applied to data analysis and follow-up information collection and did not replace routine clinical informed consent for systemic treatment. Off-label use of ADC+ICI, including use in patients without HER2 IHC 2+/3+ expression, was based on individualized clinical judgement after MDT discussion and was not mandated by the present observational study.

## 3 Results

### 3.1 Patient baseline characteristics and balancing success

A total of 421 patients with study-defined high-risk UTUC were included in the final analysis. Cohort A consisted of 320 patients treated between 2012 and 2021, including 258 patients managed with RNU alone and 62 patients receiving RNU plus adjuvant GC. Cohort B consisted of 101 patients treated between 2022 and 2025, including 48 patients receiving adjuvant GC and 53 patients receiving adjuvant ADC+ICI.

In Cohort A, patients selected for adjuvant GC were younger and had better baseline renal function than those managed with RNU alone, reflecting treatment-selection patterns in routine practice. After 1:2 propensity score matching, 104 patients in the RNU-alone group and 57 patients in the RNU+GC group were retained. Five GC-treated patients could not be matched within the prespecified caliper and were therefore excluded from the matched analysis. Baseline covariates were well balanced after matching, with all standardized mean differences below 0.10 (**Tables 1**, **2**; **Supplementary Figure 2**).

**Table 1:** Baseline clinicopathologic characteristics of study-defined high-risk upper tract urothelial carcinoma in cohorts A and B before propensity score matching/overlap weighting.

Variable	Cohort A pre-matching					Cohort B pre-weighting			
		RNU n=258(%)	RNU+GC n=62(%)	P-value	SMD	RNU+GC n=48(%)	RNU+ADC+ICI n=53(%)	P-value	SMD
Sex	Male	134(51.9)	35(56.5)	0.619	0.091	36 (75.0)	28 (52.8)	0.036	0.474
	Female	124(48.1)	27(43.5)			12 (25.0)	25 (47.2)		
Age(IQR)		68.00 [60.00,73.00]	62.00 [55.00,67.00]	<0.001	0.678	61.50 [57.75, 68.00]	66.00 [59.00, 75.00]	0.018	0.487
BMI(IQR)		23.52 [21.23,25.81]	23.85 [22.20,26.37]	0.262	0.16	23.91 [22.21, 25.71]	23.44 [21.72, 25.39]	0.612	0.129
Symptom	Hematuria	181(70.2)	41(66.1)	0.147	0.274	32 (66.7)	33 (62.3)	0.777	0.142
	Flank pain	42(16.3)	16(25.8)			11 (22.9)	12 (22.6)		
	Negative	35(13.6)	5(8.1)			5 (10.4)	8 (15.1)		
Diabetes	No	234(90.7)	58(93.5)	0.643	0.106	46 (95.8)	51 (96.2)	1.000	0.020
	Yes	24(9.3)	4(6.5)			2 (4.2)	2 (3.8)		
Hypertension	No	191(74.0)	44(71.0)	0.741	0.069	35 (72.9)	32 (60.4)	0.262	0.268
	Yes	67(26.0)	18(29.0)			13 (27.1)	21 (39.6)		
Hydronephrosis	No	111(43.0)	28(45.2)	0.871	0.043	10 (20.8)	14 (26.4)	0.671	0.132
	Yes	147(57.0)	34(54.8)			38 (79.2)	39 (73.6)		
Lymph node	pN0/pNx	226(87.6)	55(88.7)	0.981	0.034	38 (79.2)	38 (71.7)	0.524	0.174
	pN+	32(12.4)	7(11.3)			10 (20.8)	15 (28.3)		
T-stage	<T2	34(13.2)	10(16.1)	0.689	0.084	9 (18.8)	4 (7.5)	0.167	0.336
	>=T2	224(86.8)	52(83.9)			39 (81.2)	49 (92.5)		
Location	Pelvis	141(54.7)	33(53.2)	0.226	0.217	22 (45.8)	25 (47.2)	0.049	0.509
	Ureter	106(41.1)	23(37.1)			22 (45.8)	15 (28.3)		
	Multifocality	11(4.3)	6(9.7)			4 (8.3)	13 (24.5)		
Size(IQR)		3.00 [2.50,4.50]	3.25 [2.08,5.00]	0.433	0.155	3.50 [2.50, 5.00]	3.50 [2.50, 5.50]	0.665	0.168
Side	left	130(50.4)	33(53.2)	0.795	0.057	26 (54.2)	30 (56.6)	0.964	0.049
	right	128(49.6)	29(46.8)			22 (45.8)	23 (43.4)		
Grade	low	38(14.7)	6(9.7)	0.406	0.155	4 (8.3)	3 (5.7)	0.892	0.105
	high	220(85.3)	56(90.3)			44 (91.7)	50 (94.3)		
Margin	No	248(96.1)	58(93.5)	0.586	0.117	46 (95.8)	52 (98.1)	0.931	0.133
	Yes	10(3.9)	4(6.5)			2 (4.2)	1 (1.9)		
LVI	No	206(79.8)	48(77.4)	0.803	0.059	33 (68.8)	34 (64.2)	0.781	0.098
	Yes	52(20.2)	14(22.6)			15 (31.2)	19 (35.8)		
Bladder cancer	No	217(84.1)	51(82.3)	0.871	0.049	44 (91.7)	49 (92.5)	1.000	0.029
	Yes	41(15.9)	11(17.7)			4 (8.3)	4 (7.5)		
Ureteroscope	No	206(79.8)	53(85.5)	0.404	0.149	45 (93.8)	48 (90.6)	0.824	0.119
	Yes	52(20.2)	9(14.5)			3 (6.2)	5 (9.4)		
Instillation	No	127(49.2)	28(45.2)	0.665	0.081	26 (54.2)	37 (69.8)	0.157	0.327
	Yes	131(50.8)	34(54.8)			22 (45.8)	16 (30.2)		
HB(IQR)		133.00 [117.25,147.00]	133.50 [118.25,146.75]	0.688	0.065	128.00 [118.75, 142.25]	132.00 [115.00, 144.00]	0.632	0.118
eGFR (IQR)		71.25 [54.44,84.78]	76.27 [59.97,93.56]	0.012	0.312	72.55 [61.56, 90.34]	75.62 [59.16, 91.38]	0.957	0.048
SII(IQR)		478.69 [312.77,813.95]	482.18 [336.47,949.04]	0.461	0.001	509.34 [332.70, 998.60]	603.63 [367.71,1290.37]	0.492	0.034
Surgery year (IQR)		2018.00 [2016.00,2019.75]	2019.00 [2016.25,2020.00]	0.047	0.244	2023.00 [2022.00,2024.00]	2024.00 [2023.00,2024.00]	0.036	0.438
HER2	IHC 0					21 (43.8)	18 (34.0)	0.577	0.210
	IHC 1+					11 (22.9)	13 (24.5)		
	IHC 2+/3+					16 (33.3)	22 (41.5)		
ECOG	0					37 (77.1)	31 (58.5)	0.020	0.669
	1					9 (18.8)	8 (15.1)
	2					2 (4.2)	9 (17.0)
	3					0 (0.0)	5 (9.4)
partial cisplatin-ineligibility by eGFR/ECOG	No by eGFR/ECOG only					36 (75.0)	31 (58.5)	0.123	0.356
	Yes by eGFR/ECOG only					12 (25.0)	22 (41.5)		

Data are presented as n (%) or median (interquartile range), as appropriate.

ADC+ICI, disitamab vedotin plus PD-1 inhibitor; eGFR, estimated glomerular filtration rate, derived from serum creatinine using the standard equation routinely applied in our clinical laboratory; GC, gemcitabine/cisplatin; RNU, radical nephroureterectomy; SII, systemic immune–inflammation index, calculated as platelet count × neutrophil count/lymphocyte count; UTUC, upper tract urothelial carcinoma.

**Table 2:** Baseline characteristics of cohort A after propensity score matching and cohort B after overlap weighting.

Variable		Cohort A after matching				Cohort B after overlap weighting			
		RNU n=104(%)	RNU+GC n=57(%)	P-value	SMD	RNU+GC weighted %	RNU+ADC+ICI weighted %	P-value	SMD
Sex	Male	60(57.7)	32(56.1)	0.981	0.031	62.2	62.1	0.998	0.001
	Female	44(42.3)	25(43.9)			37.8	37.9		
Age(IQR)		62.00 [55.00,68.00]	64.00 [55.00,67.00]	0.956	0.022	62.00 [58.69, 67.93]	62.00 [56.00, 67.56]	0.967	0.001
BMI(IQR)		23.78 [21.33,25.86]	23.83 [21.88,25.73]	0.748	0.052	23.71 [21.94, 25.70]	23.52 [22.15, 25.47]	0.934	0.001
Symptom	Hematuria	71(68.3)	39(68.4)	0.999	0.007	56.6	56.5	1.000	0.001
	Flank pain	24(23.1)	13(22.8)			29.1	29.1		
	Negative	9(8.7)	5(8.8)			14.3	14.4		
Diabetes	No	95(91.3)	53(93.0)	0.951	0.061	97.7	97.7	0.999	0.001
	Yes	9(8.7)	4(7.0)			2.3	2.3		
Hypertension	No	75(72.1)	39(68.4)	0.755	0.081	68.2	68.2	0.998	0.001
	Yes	29(27.9)	18(31.6)			31.8	31.8		
Hydronephrosis	No	46(44.2)	25(43.9)	1.000	0.007	16.4	16.4	0.999	0.001
	Yes	58(55.8)	32(56.1)			83.6	83.6		
Lymph node	pN0/pNx	89(85.6)	50(87.7)	0.890	0.063	77.0	77.0	0.998	0.001
	pN+	15(14.4)	7(12.3)			23.0	23.0		
T-stage	<T2	17(16.3)	9(15.8)	1.000	0.015	12.0	12.0	0.992	0.003
	>=T2	87(83.7)	48(84.2)			88.0	88.0		
Location	Pelvis	58(55.8)	30(52.6)	0.923	0.066	49.8	49.7	1.000	0.001
	Ureter	38(36.5)	22(38.6)			39.3	39.3		
	Multifocality	8(7.7)	5(8.8)			10.9	10.9		
Size(IQR)		3.00 [2.50,4.62]	3.00 [2.00,5.00]	0.814	0.040	4.00 [2.60, 5.00]	3.69 [2.18, 5.00]	0.652	0.001
Side	left	57(54.8)	31(54.4)	1.000	0.008	61.8	61.8	0.998	0.001
	right	47(45.2)	26(45.6)			38.2	38.2		
Grade	low	11(10.6)	6(10.5)	1.000	0.002	5.6	5.6	0.999	0.001
	high	93(89.4)	51(89.5)			94.4	94.4		
Margin	No	98(94.2)	54(94.7)	1.000	0.022	95.4	95.4	0.999	0.001
	Yes	6(5.8)	3(5.3)			4.6	4.6		
LVI	No	81(77.9)	44(77.2)	1.000	0.017	67.4	67.3	0.998	0.001
	Yes	23(22.1)	13(22.8)			32.6	32.7		
Bladder cancer	No	88(84.6)	48(84.2)	1.000	0.011	96.2	96.2	0.999	0.001
	Yes	16(15.4)	9(15.8)			3.8	3.8		
Ureteroscope	No	85(81.7)	48(84.2)	0.857	0.066	91.2	91.2	0.999	0.001
	Yes	19(18.3)	9(15.8)			8.8	8.8		
Instillation	No	46(44.2)	26(45.6)	0.998	0.028	70.3	70.3	0.998	0.001
	Yes	58(55.8)	31(54.4)			29.7	29.7	0.998	
HB(IQR)		136.00 [123.50,149.25]	133.00 [118.00,148.00]	0.633	0.019	132.39 [121.00, 142.00]	130.61 [117.50,145.00]	0.962	0.003
eGFR(IQR)		75.97 [61.20,91.87]	71.57 [58.85,91.80]	0.828	0.020	74.93 [63.70, 90.60]	73.93 [60.82, 92.39]	0.953	0.003
SII(IQR)		472.26 [338.54,756.49]	472.28 [332.38,922.47]	0.862	0.042	505.19 [346.08, 873.85]	603.23 [416.66,1378.42]	0.330	0.001
Surgery year(IQR)		2018.00 [2017.00,2020.00]	2019.00 [2016.00,2020.00]	0.547	0.066	2023.00 [2022.72,2024.00]	2023.00 [2023.00,2024.00]	0.952	0.001
HER2	IHC 0	–				39.9	40.0	1.000	0.001
	IHC 1+					22.6	22.5		
	IHC 2+/3+					37.5	37.5		
ECOG	0					77.0	77.0	1.000	0.001
	1					14.8	14.8
	2					8.2	8.2
	3					0.0	0.0
partial cisplatin-ineligibility by eGFR/ECOG	No by eGFR/ECOG only					79.9	75.7	0.691	0.098
	Yes by eGFR/ECOG only					20.1	24.3		

Data are presented as weighted or matched n (%) or median (interquartile range), unless otherwise specified.

Cohort A: patients treated with RNU alone versus RNU plus adjuvant GC after 1:2 propensity score matching.

Cohort B: patients treated with adjuvant GC versus ADC+ICI in the overlap-weighted pseudo-population. In cohort B, values are presented as overlap-weighted percentages. Because overlap weighting creates a pseudo-population, weighted “counts” are not integers and are therefore not reported.

ADC+ICI, disitamab vedotin plus PD-1 inhibitor; eGFR, estimated glomerular filtration rate, derived from serum creatinine using the standard equation routinely applied in our clinical laboratory; GC, gemcitabine/cisplatin; HER2, human epidermal growth factor receptor 2; OW, overlap weighting; PSM, propensity score matching; RNU, radical nephroureterectomy; SII, systemic immune–inflammation index, calculated as platelet count × neutrophil count/lymphocyte count; SMD, standardized mean difference.

In Cohort B, baseline imbalances were observed before overlap weighting. Compared with the GC group, patients receiving ADC+ICI were older, had a higher proportion of female patients, worse ECOG performance status, later treatment year, and a higher proportion of partial cisplatin-ineligibility based on eGFR/ECOG criteria. ECOG performance status showed the largest imbalance before weighting. HER2 expression was reclassified as IHC 0, IHC 1+, and IHC 2+/3+. Before weighting, IHC 2+/3+ tumors were observed in 16/48 patients (33.3%) in the GC group and 22/53 patients (41.5%) in the ADC+ICI group. After overlap weighting, measured baseline covariates were well balanced, with standardized mean differences at or below 0.10. The weighted distributions of ECOG performance status, HER2 IHC category, treatment year, renal function, and pathological variables were closely aligned between groups (**Tables 1**, **2**; **Supplementary Figure 2**).

### 3.2 Cohort A: long-term survival benefit of adjuvant GC

**Figure 1:**
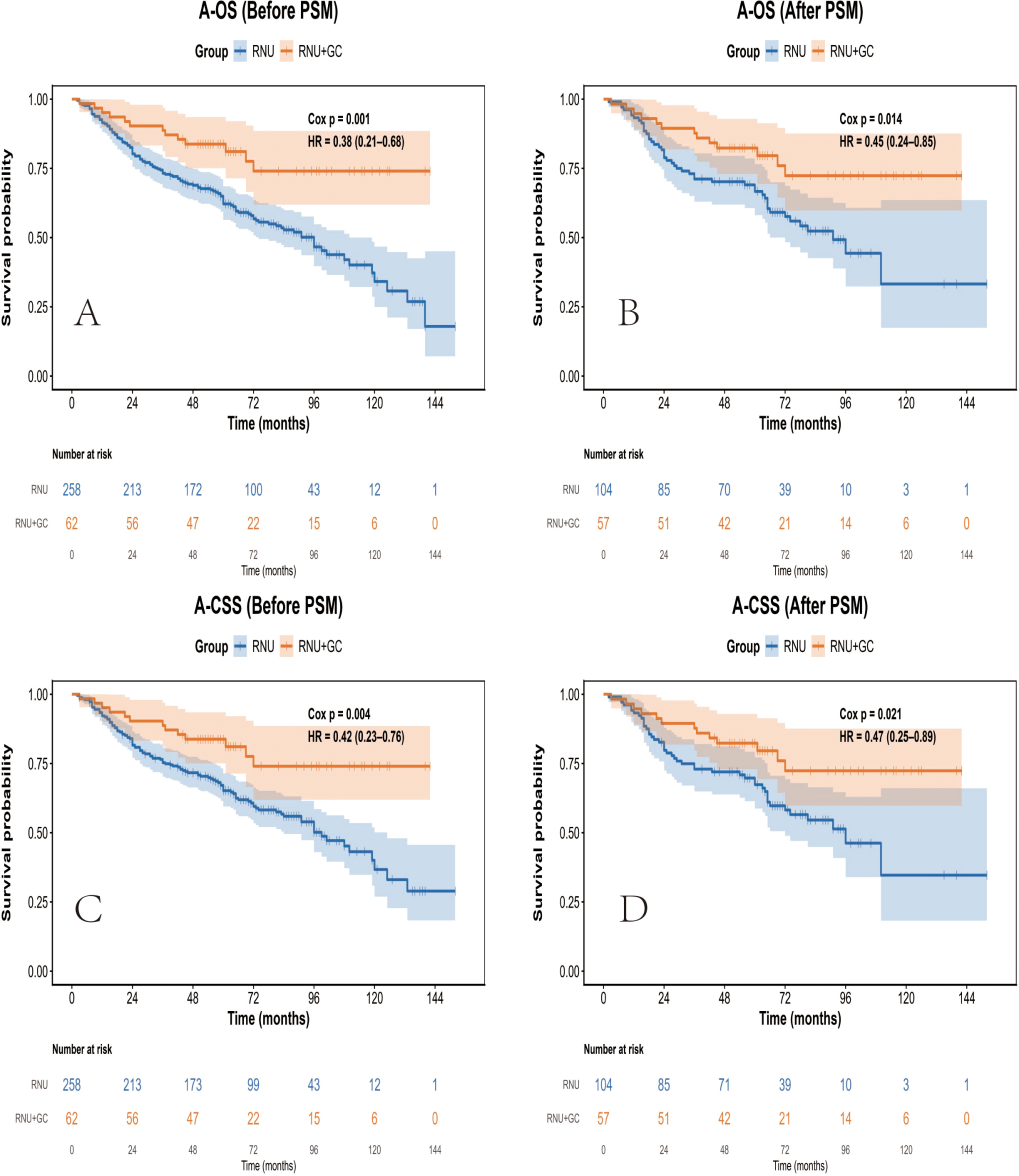
Overall and cancer-specific survival in cohort A before and after propensity score matching. **(A)** Kaplan–Meier curves for overall survival (OS) comparing RNU alone with RNU plus adjuvant GC before propensity score matching. **(B)** Kaplan–Meier curves for OS comparing RNU alone with RNU plus adjuvant GC after propensity score matching. **(C)** Kaplan–Meier curves for cancer-specific survival (CSS) comparing RNU alone with RNU plus adjuvant GC before propensity score matching. **(D)** Kaplan–Meier curves for CSS comparing RNU alone with RNU plus adjuvant GC after propensity score matching. CSS, cancer-specific survival; GC, gemcitabine/cisplatin; OS, overall survival; PSM, propensity score matching; RNU, radical nephroureterectomy.

In the propensity score–matched Cohort A, adjuvant GC was associated with improved long-term survival compared with RNU alone. The 5-year OS rate was 82.3% in the RNU+GC group and 66.7% in the RNU-alone group. In Cox regression, adjuvant GC was associated with a lower risk of all-cause mortality (HR 0.45, 95% CI 0.24–0.85; P = 0.014). A similar association was observed for CSS, with 5-year CSS rates of 82.3% versus 67.3% and an HR of 0.47 (95% CI 0.25–0.89; P = 0.021). Restricted mean survival time analysis over 60 months showed an absolute OS gain of 5.56 months associated with adjuvant GC (95% CI 0.29–10.84; P = 0.039). These findings supported the use of Cohort A as an internal reference confirming the expected benefit of platinum-based adjuvant chemotherapy in the study population (**Figure 1****;** **Supplementary Tables 1**-**4****;** **Supplementary Figures 3**,**4**).

### 3.3 Treatment exposure and completion in Cohort B

In Cohort B, treatment exposure and completion were broadly comparable between groups. The median interval from RNU to initiation of systemic adjuvant therapy was 5.7 weeks in the GC group and 6.6 weeks in the ADC+ICI group. The median number of administered cycles/doses was four in both groups. Completion of at least four cycles was achieved in 30/48 patients (62.5%) receiving GC and 35/53 patients (66.0%) receiving ADC+ICI, while completion of at least six cycles was achieved in 18/48 (37.5%) and 14/53 (26.4%) patients, respectively. Eight patients in the GC group (16.7%) switched from cisplatin to carboplatin during treatment.

Early discontinuation before four cycles occurred in 18/48 patients (37.5%) in the GC group and 18/53 patients (34.0%) in the ADC+ICI group. Among patients who discontinued early, the most common reason was patient decision or financial considerations, observed in 11/18 patients (61.1%) in the GC group and 12/18 patients (66.7%) in the ADC+ICI group. Toxicity or intolerance accounted for early discontinuation in 2/18 patients (11.1%) in the GC group and in no patients in the ADC+ICI group. These findings suggested that early discontinuation was driven mainly by non-toxicity-related factors in both groups (**Supplementary Table 7****).** Within the ADC+ICI group, 28 patients (52.8%) received toripalimab and 25 patients (47.2%) received tislelizumab.

### 3.4 Cohort B: overlap-weighted comparative outcomes

The median follow-up estimated by reverse Kaplan–Meier analysis was 80 months (IQR, 60–98) in the matched Cohort A and 20 months (IQR, 13–28) in Cohort B; after overlap weighting, the median follow-up in Cohort B was 20 months (IQR, 13–26). During follow-up in Cohort B, OS events occurred in 5/48 patients (10.4%) in the GC group and 4/53 patients (7.5%) in the ADC+ICI group. CSS events occurred in 4/48 (8.3%) and 3/53 (5.7%) patients, respectively. Conventional DFS events occurred in 13/48 (27.1%) patients receiving GC and 9/53 (17.0%) patients receiving ADC+ICI. Non-intravesical PFS events occurred in 11/48 (22.9%) and 6/53 (11.3%) patients, respectively. IVRFS events were observed in 7/48 (14.6%) patients in the GC group and 2/53 (3.8%) patients in the ADC+ICI group.

After overlap weighting, the effective sample sizes were 29.5 in the GC group and 29.1 in the ADC+ICI group. ADC+ICI was not associated with a statistically significant OS improvement compared with GC (HR 0.47, 95% CI 0.12–1.89; P = 0.287; FDR-adjusted P = 0.403). Directionally favorable associations were observed for conventional DFS (HR 0.32, 95% CI 0.11–0.96; nominal P = 0.043; FDR-adjusted P = 0.172), non-intravesical PFS (HR 0.32, 95% CI 0.09–1.18; P = 0.086), and IVRFS (HR 0.21, 95% CI 0.04–1.09; P = 0.063). CSS and MFS did not differ significantly between groups.

At 24 months, overlap-weighted OS was 93.8% in the ADC+ICI group and 82.0% in the GC group. The corresponding 24-month conventional DFS, non-intravesical PFS, and IVRFS rates were 83.0% versus 52.7%, 91.6% versus 73.3%, and 97.6% versus 83.8%, respectively. Given the limited number of OS and CSS events and the small effective sample size after weighting, these estimates were interpreted as preliminary.

**Figure 2:**
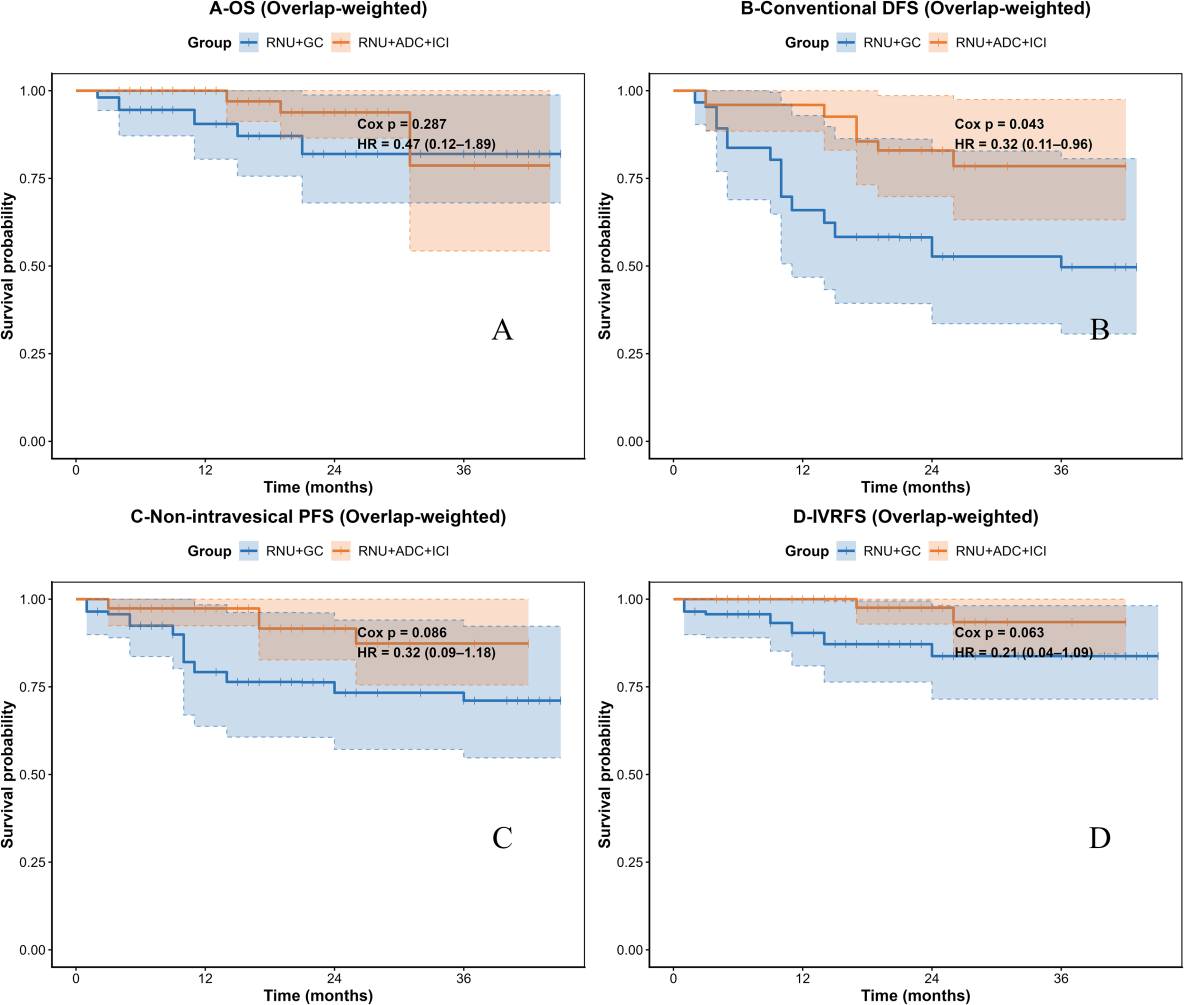
Overlap-weighted survival outcomes comparing adjuvant GC with ADC+ICI in Cohort B. Curves were generated after overlap weighting. **(A)** Overall survival. **(B)** Conventional disease-free survival. **(C)** Non-intravesical progression-free survival. **(D)** Intravesical recurrence-free survival. Original sample sizes and effective sample sizes after overlap weighting are reported in **Supplementary Table 5**. ADC+ICI, disitamab vedotin plus PD-1 inhibitor; DFS, disease-free survival; GC, gemcitabine/cisplatin; IVRFS, intravesical recurrence-free survival; OS, overall survival; PFS, progression-free survival.

In a sensitivity analysis excluding GC patients who switched from cisplatin to carboplatin, the favorable associations observed in the main overlap-weighted analysis were attenuated and did not reach statistical significance. The HRs were 1.29 for OS (95% CI 0.29–5.80; P = 0.738), 0.74 for DFS (95% CI 0.31–1.74; P = 0.484), and 0.51 for non-intravesical PFS (95% CI 0.20–1.35; P = 0.176). These findings suggest that platinum fitness and treatment-selection factors may partly influence the observed comparative outcomes (**Figure 2****;** **Supplementary Tables 5**-**7**).

### 3.5 Exploratory HER2-stratified analyses

HER2 expression was reclassified into three categories: IHC 0, IHC 1+, and IHC 2+/3+. In the GC group, 21 patients (43.8%) had IHC 0, 11 (22.9%) had IHC 1+, and 16 (33.3%) had IHC 2+/3+ tumors. In the ADC+ICI group, the corresponding numbers were 18 (34.0%), 13 (24.5%), and 22 (41.5%), respectively.

**Figure 3:**
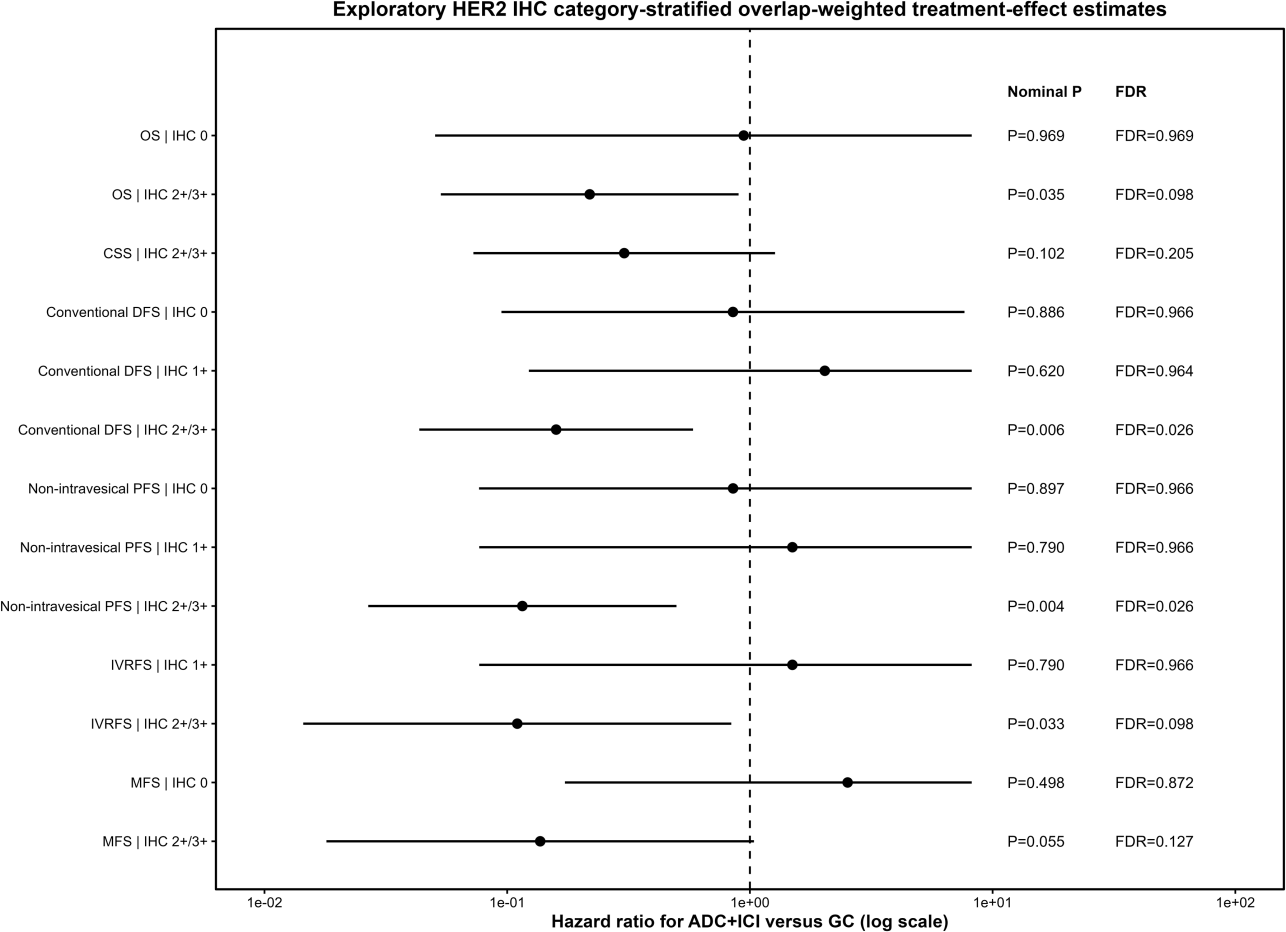
Exploratory HER2-stratified treatment-effect estimates in Cohort B. Hazard ratios for ADC+ICI versus GC are shown across HER2 IHC 0, IHC 1+, and IHC 2+/3+ strata. These analyses were exploratory and underpowered; formal treatment-by-HER2 interaction testing was limited by sparse event counts. ADC+ICI, disitamab vedotin plus PD-1 inhibitor; DFS, disease-free survival; FDR, false discovery rate; GC, gemcitabine/cisplatin; HER2, human epidermal growth factor receptor 2; IHC, immunohistochemistry; PFS, progression-free survival.

In exploratory HER2-stratified analyses, treatment-effect estimates appeared more favorable among patients with HER2 IHC 2+/3+ tumors. In this subgroup, ADC+ICI was associated with lower hazards of conventional DFS events (HR 0.16, 95% CI 0.04–0.58; P = 0.006; FDR-adjusted P = 0.026) and non-intravesical PFS events (HR 0.12, 95% CI 0.03–0.50; P = 0.004; FDR-adjusted P = 0.026). However, the associations for OS and IVRFS were less robust after multiplicity adjustment, and formal treatment-by-HER2 interaction testing was not considered reliable for several endpoints because of sparse event counts within HER2 strata. Therefore, these analyses should be interpreted as exploratory and hypothesis-generating rather than confirmatory evidence of a predictive biomarker effect (**Figure 3****;** **Supplementary Table 8**).

### 3.6 Safety and treatment-related adverse events

The overall incidence of any treatment-related adverse event was 33/48 (68.8%) in the GC group and 31/53 (58.5%) in the ADC+ICI group (P = 0.285). The incidence of grade ≥3 treatment-related adverse events was similar between groups, occurring in 4/48 patients (8.3%) receiving GC and 4/53 patients (8.0%) receiving ADC+ICI (P = 1.000).

The toxicity profiles differed between regimens. Nausea/vomiting was more frequent in the GC group than in the ADC+ICI group (43.8% vs 20.8%; P = 0.013), as was myelosuppression (12.5% vs 0%; P = 0.010). In contrast, peripheral neuropathy was more frequent in the ADC+ICI group (15.1% vs 2.1%; P = 0.033), although most cases were low grade and clinically manageable. Treatment discontinuation due to toxicity occurred in 2/48 patients (4.2%) receiving GC and in no patients receiving ADC+ICI (P = 0.220). No treatment-related deaths were observed (**Table 3**).

**Table 3:** Treatment-related adverse events in Cohort B.

AE	RNU+GC (n=48)	RNU+ADC+ICI (n=53)	P-value
Any TRAE	33 (68.8%)	31 (58.5%)	0.285
Nausea/vomiting	21 (43.8%)	11 (20.8%)	0.013
Myelosuppression	6 (12.5%)	0 (0.0%)	0.010
Neuropathy	1 (2.1%)	8 (15.1%)	0.033
Fatigue	4 (8.3%)	4 (7.5%)	1.000
Alopecia	8 (16.7%)	4 (7.5%)	0.157
Rash	1 (2.1%)	5 (9.4%)	0.208
Hyperglycemia	0 (0.0%)	3 (5.7%)	0.244
Edema	0 (0.0%)	1 (1.9%)	1.000
Any grade ≥3 TRAE	4 (8.3%)	4 (8.0%)	1.000
Discontinuation due to Toxicity	2 (4.2%)	0 (0.0%)	0.220

Data are presented as n (%). Adverse events were graded according to the Common Terminology Criteria for Adverse Events (CTCAE), version 5.0.

ADC+ICI, disitamab vedotin plus PD-1 inhibitor; GC, gemcitabine/cisplatin; TRAE, treatment-related adverse event.

## 4 Discussion

The management of high-risk upper tract urothelial carcinoma (UTUC) after radical nephroureterectomy remains challenging, particularly because postoperative renal function decline and treatment tolerance may limit the use of standard cisplatin-based adjuvant chemotherapy. In this single-centre, two-stage real-world study, Cohort A reaffirmed the expected association between adjuvant gemcitabine/cisplatin (GC) and improved long-term survival compared with surgery alone, whereas Cohort B provided exploratory comparative data for adjuvant disitamab vedotin plus PD-1 blockade (ADC+ICI) versus GC in contemporary clinical practice. After overlap weighting with updated adjustment for ECOG performance status, HER2 IHC category, treatment year, renal function, and other measured covariates, ADC+ICI showed comparable short-term OS and CSS to GC, with directionally favorable associations for conventional DFS, non-intravesical PFS, and IVRFS. However, the OS comparison was not statistically significant, and the nominal DFS association did not remain significant after FDR adjustment. Therefore, the Cohort B findings should be interpreted as preliminary and hypothesis-generating rather than as definitive evidence that ADC+ICI is superior to GC (12, 13).

The results from Cohort A are consistent with the established role of platinum-based adjuvant chemotherapy after RNU in appropriately selected patients. In the matched analysis, adjuvant GC was associated with improved OS and CSS compared with RNU alone. This finding supports the internal validity of the study population and is broadly consistent with the POUT trial, which established platinum-based adjuvant chemotherapy as a standard postoperative option for high-risk UTUC. Nevertheless, the real-world treatment pattern in Cohort A also illustrates an important clinical issue: patients selected for adjuvant GC were generally younger and had better baseline renal function before matching. This reflects confounding by indication and highlights that cisplatin-based chemotherapy is preferentially administered to fitter patients with adequate postoperative renal reserve (6, 14).

Cohort B was designed to explore whether ADC+ICI could represent a feasible postoperative option in a contemporary high-risk population in which platinum suitability is often uncertain. Before weighting, patients receiving ADC+ICI were older, had worse ECOG performance status, and included a higher proportion of patients classified as partially cisplatin-ineligible based on eGFR/ECOG criteria. Overlap weighting substantially improved measured covariate balance, but it cannot replicate randomization or fully account for unmeasured determinants of treatment selection. Importantly, when GC patients who switched from cisplatin to carboplatin were excluded, the favorable associations observed in the main analysis were attenuated and no longer statistically significant. This finding supports a cautious interpretation and suggests that platinum fitness and treatment-selection factors may partly influence the observed comparative outcomes.

The clinical relevance of ADC+ICI in this setting should therefore be viewed in terms of feasibility, tolerability, and exploratory disease-control signals rather than confirmed survival benefit. In Cohort B, OS and CSS events were infrequent, and median survival was not reached in either group. The effective sample size after overlap weighting was also limited. For these reasons, estimates beyond 24 months, especially 3-year OS estimates, are unstable and should not be overinterpreted. The lower numerical incidence of DFS, non-intravesical PFS, and IVRFS events in the ADC+ICI group is clinically interesting, but requires validation in larger prospective cohorts with longer follow-up (15, 16).

HER2 expression is biologically relevant to disitamab vedotin, but our findings do not establish HER2 as a validated predictive biomarker in the adjuvant UTUC setting. In the revised analysis, HER2 status was reclassified as IHC 0, IHC 1+, and IHC 2+/3+ to avoid grouping HER2-low tumors with HER2-null disease. The exploratory subgroup analysis suggested that patients with IHC 2+/3+ tumors may derive a more favorable DFS and non-intravesical PFS signal from ADC+ICI. However, the number of events within HER2 strata was small, the OS and IVRFS findings were less robust after multiplicity adjustment, and formal treatment-by-HER2 interaction testing was not reliable for several endpoints because of sparse events. Therefore, the HER2-stratified results should be regarded as hypothesis-generating and useful primarily for informing future biomarker-enriched study designs (17, 18).

The toxicity profile observed in Cohort B also has practical implications for postoperative treatment selection. The overall incidence of treatment-related adverse events and grade ≥3 adverse events was similar between groups, but the pattern of toxicity differed. GC was associated with higher rates of nausea/vomiting and myelosuppression, whereas ADC+ICI was associated with more peripheral neuropathy. Treatment discontinuation due to toxicity was uncommon in both groups, and early discontinuation was driven mainly by patient decision, financial considerations, logistics, or transfer of care. These findings suggest that ADC+ICI is clinically feasible in selected postoperative patients, but the risk of neuropathy should be carefully monitored, particularly because neuropathy may affect quality of life in the adjuvant setting (9, 19).

Several limitations should be emphasized. First, this was a single-centre, non-randomized observational study, and treatment allocation in Cohort B was influenced by MDT decision-making, postoperative renal function, ECOG performance status, HER2 expression, comorbidities, drug availability, financial considerations, and patient preference. Although overlap weighting achieved good balance across measured covariates, residual confounding remains possible. Second, full Galsky-defined cisplatin eligibility could not be reconstructed because baseline hearing impairment, pre-existing peripheral neuropathy grade, and NYHA cardiac function were not uniformly recorded. The partial cisplatin-ineligibility variable used in this study was therefore based only on eGFR and ECOG performance status. Third, Cohort B survival data remain immature, with a limited number of OS and CSS events and a small effective sample size after weighting. In addition, the present cohort did not evaluate an optimized strategy of ADC+ICI induction followed by long-term PD-1 maintenance; therefore, our findings cannot determine the efficacy of maintenance-based adjuvant immunotherapy. Fourth, PD-L1 CPS was not routinely available and could not be incorporated into treatment allocation adjustment or subgroup analysis. Fifth, HER2- and PD-1 inhibitor–stratified analyses were exploratory and underpowered. The inclusion of patients with HER2 IHC 0/1+ tumors in the ADC+ICI group reflects individualized off-label use in routine clinical practice and should not be interpreted as evidence supporting a regulatory indication or standard adjuvant use of disitamab vedotin in HER2 IHC 0/1+ disease. Finally, because the study excluded patients who received neoadjuvant systemic therapy, the findings apply only to patients undergoing post-RNU adjuvant decision-making and should not be generalized to all patients with high-risk UTUC.

In conclusion, this two-stage real-world study supports the established role of adjuvant GC in appropriately selected high-risk UTUC patients and provides preliminary evidence that adjuvant disitamab vedotin plus PD-1 blockade is a feasible postoperative treatment option with a distinct toxicity profile. Compared with GC, ADC+ICI showed comparable short-term OS and directionally favorable DFS/PFS-related trends, but definitive superiority was not demonstrated. These findings should be considered hypothesis-generating and warrant validation in larger, multicenter, prospective studies with longer follow-up and systematic assessment of cisplatin eligibility, HER2 status, and PD-L1 expression.

## Data Availability

The datasets generated and/or analyzed during the current study are not publicly available due to institutional data-protection policies and the inclusion of patient-level clinical data, but are available from the corresponding author on reasonable request and subject to institutional approval.
