# Supplementary material for: Adjuvant disitamab vedotin plus PD-1 blockade and gemcitabine/cisplatin in high-risk upper tract urothelial carcinoma: a two-stage real-world comparative study
Source: Front Immunol. 2026 Jun 16;17:1809094. doi: 10.3389/fimmu.2026.1809094 (PMC13315034; doi:10.3389/fimmu.2026.1809094)
Supplement: Supplementary file 1 [file SupplementaryFile1.docx]

Supporting Information for:

Adjuvant Disitamab Vedotin Plus PD-1 Blockade and Gemcitabine/Cisplatin in High-Risk Upper Tract Urothelial Carcinoma: A Two-Stage Real-World Comparative Study

This file includes:

Supplementary Tables 1–9

Supplementary Figures 1–5

**Supplementary Table 1.** Kaplan–Meier estimated 1-, 3-, and 5-year OS, CSS, non-intravesical PFS, IVRFS, and MFS in Cohort A before and after propensity score matching

| Endpoint | Period | Time | Group | Survival | LCI | UCI |
| --- | --- | --- | --- | --- | --- | --- |
| OS | Before PSM | 12 | RNU | 0.915 | 0.881 | 0.949 |
| OS | Before PSM | 36 | RNU | 0.732 | 0.68 | 0.788 |
| OS | Before PSM | 60 | RNU | 0.622 | 0.564 | 0.685 |
| OS | Before PSM | 12 | RNU+GC | 0.952 | 0.9 | 1 |
| OS | Before PSM | 36 | RNU+GC | 0.887 | 0.812 | 0.969 |
| OS | Before PSM | 60 | RNU+GC | 0.838 | 0.751 | 0.935 |
| OS | After PSM | 12 | RNU | 0.933 | 0.886 | 0.982 |
| OS | After PSM | 36 | RNU | 0.712 | 0.63 | 0.804 |
| OS | After PSM | 60 | RNU | 0.667 | 0.581 | 0.765 |
| OS | After PSM | 12 | RNU+GC | 0.947 | 0.891 | 1 |
| OS | After PSM | 36 | RNU+GC | 0.877 | 0.796 | 0.967 |
| OS | After PSM | 60 | RNU+GC | 0.823 | 0.73 | 0.929 |
| non-intravesical PFS | Before PSM | 12 | RNU | 0.841 | 0.797 | 0.888 |
| non-intravesical PFS | Before PSM | 36 | RNU | 0.732 | 0.679 | 0.79 |
| non-intravesical PFS | Before PSM | 60 | RNU | 0.658 | 0.599 | 0.722 |
| non-intravesical PFS | Before PSM | 12 | RNU+GC | 0.855 | 0.771 | 0.947 |
| non-intravesical PFS | Before PSM | 36 | RNU+GC | 0.755 | 0.654 | 0.871 |
| non-intravesical PFS | Before PSM | 60 | RNU+GC | 0.717 | 0.611 | 0.841 |
| non-intravesical PFS | After PSM | 12 | RNU | 0.853 | 0.787 | 0.925 |
| non-intravesical PFS | After PSM | 36 | RNU | 0.716 | 0.632 | 0.812 |
| non-intravesical PFS | After PSM | 60 | RNU | 0.653 | 0.562 | 0.758 |
| non-intravesical PFS | After PSM | 12 | RNU+GC | 0.842 | 0.752 | 0.942 |
| non-intravesical PFS | After PSM | 36 | RNU+GC | 0.733 | 0.625 | 0.858 |
| non-intravesical PFS | After PSM | 60 | RNU+GC | 0.691 | 0.579 | 0.826 |
| CSS | Before PSM | 12 | RNU | 0.922 | 0.89 | 0.955 |
| CSS | Before PSM | 36 | RNU | 0.753 | 0.702 | 0.808 |
| CSS | Before PSM | 60 | RNU | 0.652 | 0.595 | 0.715 |
| CSS | Before PSM | 12 | RNU+GC | 0.952 | 0.9 | 1 |
| CSS | Before PSM | 36 | RNU+GC | 0.887 | 0.812 | 0.969 |
| CSS | Before PSM | 60 | RNU+GC | 0.838 | 0.751 | 0.935 |
| CSS | After PSM | 12 | RNU | 0.933 | 0.886 | 0.982 |
| CSS | After PSM | 36 | RNU | 0.73 | 0.649 | 0.82 |
| CSS | After PSM | 60 | RNU | 0.673 | 0.587 | 0.772 |
| CSS | After PSM | 12 | RNU+GC | 0.947 | 0.891 | 1 |
| CSS | After PSM | 36 | RNU+GC | 0.877 | 0.796 | 0.967 |
| CSS | After PSM | 60 | RNU+GC | 0.823 | 0.73 | 0.929 |
| IVRFS | Before PSM | 12 | RNU | 0.924 | 0.891 | 0.957 |
| IVRFS | Before PSM | 36 | RNU | 0.873 | 0.832 | 0.917 |
| IVRFS | Before PSM | 60 | RNU | 0.821 | 0.77 | 0.875 |
| IVRFS | Before PSM | 12 | RNU+GC | 0.918 | 0.852 | 0.99 |
| IVRFS | Before PSM | 36 | RNU+GC | 0.885 | 0.808 | 0.969 |
| IVRFS | Before PSM | 60 | RNU+GC | 0.845 | 0.756 | 0.944 |
| IVRFS | After PSM | 12 | RNU | 0.922 | 0.871 | 0.975 |
| IVRFS | After PSM | 36 | RNU | 0.833 | 0.761 | 0.912 |
| IVRFS | After PSM | 60 | RNU | 0.771 | 0.686 | 0.868 |
| IVRFS | After PSM | 12 | RNU+GC | 0.911 | 0.839 | 0.989 |
| IVRFS | After PSM | 36 | RNU+GC | 0.874 | 0.791 | 0.966 |
| IVRFS | After PSM | 60 | RNU+GC | 0.83 | 0.733 | 0.939 |
| MFS | Before PSM | 12 | RNU | 0.909 | 0.874 | 0.945 |
| MFS | Before PSM | 36 | RNU | 0.842 | 0.797 | 0.889 |
| MFS | Before PSM | 60 | RNU | 0.823 | 0.776 | 0.873 |
| MFS | Before PSM | 12 | RNU+GC | 0.935 | 0.875 | 0.999 |
| MFS | Before PSM | 36 | RNU+GC | 0.868 | 0.786 | 0.957 |
| MFS | Before PSM | 60 | RNU+GC | 0.849 | 0.763 | 0.945 |
| MFS | After PSM | 12 | RNU | 0.911 | 0.858 | 0.968 |
| MFS | After PSM | 36 | RNU | 0.837 | 0.767 | 0.914 |
| MFS | After PSM | 60 | RNU | 0.813 | 0.739 | 0.895 |
| MFS | After PSM | 12 | RNU+GC | 0.929 | 0.865 | 0.999 |
| MFS | After PSM | 36 | RNU+GC | 0.856 | 0.768 | 0.953 |
| MFS | After PSM | 60 | RNU+GC | 0.836 | 0.743 | 0.94 |

*Footnote:*
Data are presented as estimated survival probabilities with 95% confidence intervals. The originally defined PFS endpoint was clarified as non-intravesical PFS in the revised manuscript.

CSS, cancer-specific survival; GC, gemcitabine/cisplatin; IVRFS, intravesical recurrence-free survival; MFS, metastasis-free survival; non-intravesical PFS, non-intravesical progression-free survival; OS, overall survival; PSM, propensity score matching; RNU, radical nephroureterectomy.

**Supplementary Table 2.** Propensity score–matched Cox proportional hazards models for survival endpoints in Cohort A

| Endpoint | HR | Lower 95% CI | Upper 95% CI | P |
| --- | --- | --- | --- | --- |
| OS | 0.449221 | 0.236631 | 0.852802 | 0.014412 |
| non-intravesical PFS | 0.936458 | 0.560098 | 1.565715 | 0.802325 |
| CSS | 0.47103 | 0.248628 | 0.892377 | 0.02093 |
| IVRFS | 0.766084 | 0.345784 | 1.697257 | 0.51148 |
| MFS | 0.936476 | 0.44838 | 1.955899 | 0.861344 |

*Footnote:*
Hazard ratios (HR) with 95% confidence intervals are reported for OS, CSS, non-intravesical PFS, IVRFS and MFS in the propensity score–matched cohort.
CSS, cancer-specific survival; GC, gemcitabine/cisplatin; IVRFS, intravesical recurrence-free survival; MFS, metastasis-free survival; OS, overall survival; non-intravesical PFS, non-intravesical progression-free survival; PSM, propensity score matching; RNU, radical nephroureterectomy.

**Supplementary Table 3.** Restricted mean survival time analyses for overall survival in Cohorts A and B

| Label | Tau | RMST_group0 | RMST_group1 | Diff | Lower 95% CI | Upper 95% CI | P |
| --- | --- | --- | --- | --- | --- | --- | --- |
| A After PSM (OS) | 60 | 48.15 | 53.72 | 5.56 | 0.29 | 10.84 | 0.039 |
| B OW (OS) | 44 | 39.87 | 39.47 | 0.39 | -5.90 | 5.11 | 0.887 |

*Footnote:*
Positive differences indicate longer restricted mean survival time in the treatment group. In Cohort A, the treatment group was RNU+GC; in Cohort B, the treatment group was ADC+ICI.

ADC+ICI, disitamab vedotin plus PD-1 inhibitor; GC, gemcitabine/cisplatin; OS, overall survival; OW, overlap weighting; PSM, propensity score matching; RMST, restricted mean survival time; RNU, radical nephroureterectomy.

**Supplementary Table 4.** Competing-risk sensitivity analyses for IVRFS and CSS

| Dataset | Endpoint | term | sHR | Lower 95% CI | Upper 95% CI | P |
| --- | --- | --- | --- | --- | --- | --- |
| A Before PSM | IVRFS | RNU+GC | 0.89 | 0.43 | 1.85 | 0.760 |
| A After PSM | IVRFS | RNU+GC | 0.76 | 0.35 | 1.67 | 0.500 |
| B (unweighted FG) | IVRFS | RNU+ADC+ICI | 0.28 | 0.06 | 1.38 | 0.120 |
| A Before PSM | CSS | RNU+GC | 0.43 | 0.24 | 0.77 | 0.005 |
| A After PSM | CSS | RNU+GC | 0.48 | 0.26 | 0.91 | 0.024 |
| B (unweighted FG) | CSS | RNU+ADC+ICI | 0.84 | 0.17 | 4.14 | 0.830 |

*Footnote:*
Subdistribution hazard ratios were estimated using Fine–Gray models. For IVRFS, death before intravesical recurrence was treated as a competing event. For CSS, non-cancer death was treated as a competing event. In Cohort B, competing-risk analyses were exploratory because of the limited number of events.

**Supplementary Table 5.** Event counts, effective sample size, and overlap-weighted Cox proportional hazards models for survival endpoints in Cohort B

Panel A. Event counts after overlap weighting

| Endpoint | Events in GC, n/N (%) | Events in ADC+ICI, n/N (%) | HR | 95% CI | Nominal P value | FDR-adjusted P value |
| --- | --- | --- | --- | --- | --- | --- |
| OS | 5/48 (10.4) | 4/53 (7.5) | 0.47 | 0.12–1.89 | 0.287 | 0.403 |
| CSS | 4/48 (8.3) | 3/53 (5.7) | 0.46 | 0.09–2.26 | 0.336 | 0.403 |
| Conventional DFS | 13/48 (27.1) | 9/53 (17.0) | 0.32 | 0.11–0.96 | 0.043 | 0.172 |
| Non-intravesical PFS | 11/48 (22.9) | 6/53 (11.3) | 0.32 | 0.09–1.18 | 0.086 | 0.172 |
| IVRFS | 7/48 (14.6) | 2/53 (3.8) | 0.21 | 0.04–1.09 | 0.063 | 0.172 |
| MFS | 4/48 (8.3) | 4/53 (7.5) | 0.49 | 0.08–2.91 | 0.433 | 0.433 |

Panel B. Effective sample size after overlap weighting

| Group | Original (N) | Positive-weight (N) | Effective sample size |
| --- | --- | --- | --- |
| GC | 48 | 48 | 29.5 |
| ADC+ICI | 53 | 53 | 29.1 |

*Footnote:*
Hazard ratios compare ADC+ICI with GC after overlap weighting. FDR adjustment was performed using the Benjamini–Hochberg method across the main Cohort B survival endpoints.

ADC+ICI, disitamab vedotin plus PD-1 inhibitor; CSS, cancer-specific survival; DFS, disease-free survival; FDR, false discovery rate; GC, gemcitabine/cisplatin; IVRFS, intravesical recurrence-free survival; MFS, metastasis-free survival; OS, overall survival; PFS, progression-free survival.

**Supplementary Table 6.** Kaplan–Meier estimated 12-, 18-, and 24-month survival probabilities in Cohort B before and after overlap weighting

| Endpoint | Period | Time, months | Group | Survival probability | Lower 95% CI | Upper 95% CI |
| --- | --- | --- | --- | --- | --- | --- |
| OS | Unweighted | 12 | RNU+GC | 0.934 | 0.864 | 1 |
| OS | Unweighted | 18 | RNU+GC | 0.907 | 0.824 | 0.999 |
| OS | Unweighted | 24 | RNU+GC | 0.871 | 0.768 | 0.987 |
| OS | Unweighted | 12 | RNU+ADC+ICI | 1 | 1 | 1 |
| OS | Unweighted | 18 | RNU+ADC+ICI | 0.972 | 0.92 | 1 |
| OS | Unweighted | 24 | RNU+ADC+ICI | 0.903 | 0.803 | 1 |
| Conventional DFS | Unweighted | 12 | RNU+GC | 0.767 | 0.629 | 0.934 |
| Conventional DFS | Unweighted | 18 | RNU+GC | 0.667 | 0.518 | 0.859 |
| Conventional DFS | Unweighted | 24 | RNU+GC | 0.56 | 0.393 | 0.798 |
| Conventional DFS | Unweighted | 12 | RNU+ADC+ICI | 0.893 | 0.785 | 1 |
| Conventional DFS | Unweighted | 18 | RNU+ADC+ICI | 0.75 | 0.606 | 0.929 |
| Conventional DFS | Unweighted | 24 | RNU+ADC+ICI | 0.708 | 0.556 | 0.902 |
| Non-intravesical PFS | Unweighted | 12 | RNU+GC | 0.861 | 0.764 | 0.972 |
| Non-intravesical PFS | Unweighted | 18 | RNU+GC | 0.809 | 0.698 | 0.939 |
| Non-intravesical PFS | Unweighted | 24 | RNU+GC | 0.727 | 0.589 | 0.897 |
| Non-intravesical PFS | Unweighted | 12 | RNU+ADC+ICI | 0.937 | 0.87 | 1 |
| Non-intravesical PFS | Unweighted | 18 | RNU+ADC+ICI | 0.876 | 0.778 | 0.987 |
| Non-intravesical PFS | Unweighted | 24 | RNU+ADC+ICI | 0.876 | 0.778 | 0.987 |
| CSS | Unweighted | 12 | RNU+GC | 0.934 | 0.864 | 1 |
| CSS | Unweighted | 18 | RNU+GC | 0.934 | 0.864 | 1 |
| CSS | Unweighted | 24 | RNU+GC | 0.896 | 0.802 | 1 |
| CSS | Unweighted | 12 | RNU+ADC+ICI | 1 | 1 | 1 |
| CSS | Unweighted | 18 | RNU+ADC+ICI | 0.972 | 0.92 | 1 |
| CSS | Unweighted | 24 | RNU+ADC+ICI | 0.938 | 0.857 | 1 |
| IVRFS | Unweighted | 12 | RNU+GC | 0.908 | 0.826 | 0.999 |
| IVRFS | Unweighted | 18 | RNU+GC | 0.853 | 0.75 | 0.97 |
| IVRFS | Unweighted | 24 | RNU+GC | 0.806 | 0.679 | 0.956 |
| IVRFS | Unweighted | 12 | RNU+ADC+ICI | 1 | 1 | 1 |
| IVRFS | Unweighted | 18 | RNU+ADC+ICI | 0.968 | 0.908 | 1 |
| IVRFS | Unweighted | 24 | RNU+ADC+ICI | 0.968 | 0.908 | 1 |
| MFS | Unweighted | 12 | RNU+GC | 0.953 | 0.89 | 1 |
| MFS | Unweighted | 18 | RNU+GC | 0.953 | 0.89 | 1 |
| MFS | Unweighted | 24 | RNU+GC | 0.914 | 0.823 | 1 |
| MFS | Unweighted | 12 | RNU+ADC+ICI | 0.957 | 0.899 | 1 |
| MFS | Unweighted | 18 | RNU+ADC+ICI | 0.895 | 0.801 | 1 |
| MFS | Unweighted | 24 | RNU+ADC+ICI | 0.895 | 0.801 | 1 |
| OS | OW weighted | 12 | RNU+GC | 0.905 | 0.805 | 1 |
| OS | OW weighted | 18 | RNU+GC | 0.871 | 0.756 | 1 |
| OS | OW weighted | 24 | RNU+GC | 0.82 | 0.68 | 0.988 |
| OS | OW weighted | 12 | RNU+ADC+ICI | 1 | 1 | 1 |
| OS | OW weighted | 18 | RNU+ADC+ICI | 0.97 | 0.912 | 1 |
| OS | OW weighted | 24 | RNU+ADC+ICI | 0.938 | 0.865 | 1 |
| Conventional DFS | OW weighted | 12 | RNU+GC | 0.659 | 0.468 | 0.929 |
| Conventional DFS | OW weighted | 18 | RNU+GC | 0.583 | 0.394 | 0.863 |
| Conventional DFS | OW weighted | 24 | RNU+GC | 0.527 | 0.335 | 0.828 |
| Conventional DFS | OW weighted | 12 | RNU+ADC+ICI | 0.959 | 0.884 | 1 |
| Conventional DFS | OW weighted | 18 | RNU+ADC+ICI | 0.855 | 0.732 | 1 |
| Conventional DFS | OW weighted | 24 | RNU+ADC+ICI | 0.83 | 0.698 | 0.986 |
| Non-intravesical PFS | OW weighted | 12 | RNU+GC | 0.792 | 0.638 | 0.984 |
| Non-intravesical PFS | OW weighted | 18 | RNU+GC | 0.764 | 0.607 | 0.962 |
| Non-intravesical PFS | OW weighted | 24 | RNU+GC | 0.733 | 0.572 | 0.941 |
| Non-intravesical PFS | OW weighted | 12 | RNU+ADC+ICI | 0.974 | 0.924 | 1 |
| Non-intravesical PFS | OW weighted | 18 | RNU+ADC+ICI | 0.916 | 0.827 | 1 |
| Non-intravesical PFS | OW weighted | 24 | RNU+ADC+ICI | 0.916 | 0.827 | 1 |
| CSS | OW weighted | 12 | RNU+GC | 0.905 | 0.805 | 1 |
| CSS | OW weighted | 18 | RNU+GC | 0.905 | 0.805 | 1 |
| CSS | OW weighted | 24 | RNU+GC | 0.852 | 0.719 | 1 |
| CSS | OW weighted | 12 | RNU+ADC+ICI | 1 | 1 | 1 |
| CSS | OW weighted | 18 | RNU+ADC+ICI | 0.97 | 0.912 | 1 |
| CSS | OW weighted | 24 | RNU+ADC+ICI | 0.961 | 0.901 | 1 |
| IVRFS | OW weighted | 12 | RNU+GC | 0.904 | 0.81 | 1 |
| IVRFS | OW weighted | 18 | RNU+GC | 0.872 | 0.764 | 0.995 |
| IVRFS | OW weighted | 24 | RNU+GC | 0.838 | 0.715 | 0.981 |
| IVRFS | OW weighted | 12 | RNU+ADC+ICI | 1 | 1 | 1 |
| IVRFS | OW weighted | 18 | RNU+ADC+ICI | 0.976 | 0.929 | 1 |
| IVRFS | OW weighted | 24 | RNU+ADC+ICI | 0.976 | 0.929 | 1 |
| MFS | OW weighted | 12 | RNU+GC | 0.885 | 0.739 | 1 |
| MFS | OW weighted | 18 | RNU+GC | 0.885 | 0.739 | 1 |
| MFS | OW weighted | 24 | RNU+GC | 0.884 | 0.739 | 1 |
| MFS | OW weighted | 12 | RNU+ADC+ICI | 0.974 | 0.924 | 1 |
| MFS | OW weighted | 18 | RNU+ADC+ICI | 0.932 | 0.852 | 1 |
| MFS | OW weighted | 24 | RNU+ADC+ICI | 0.932 | 0.852 | 1 |

*Footnote:*

Unweighted and overlap-weighted 12-, 18-, and 24-month survival probabilities with 95% confidence intervals are presented for OS, CSS, conventional DFS, non-intravesical PFS, IVRFS, and MFS according to adjuvant regimen. Survival probabilities are shown as proportions.

ADC+ICI, disitamab vedotin plus PD-1 inhibitor; CSS, cancer-specific survival; DFS, disease-free survival; GC, gemcitabine/cisplatin; IVRFS, intravesical recurrence-free survival; MFS, metastasis-free survival; non-intravesical PFS, non-intravesical progression-free survival; OS, overall survival; OW, overlap weighting.

**Supplementary Table 7.** Treatment exposure/completion and reasons for early discontinuation in Cohort B

Panel A. Treatment exposure and completion.

| Variable | RNU+GC (n=48) | RNU+ADC+ICI (n=53) |
| --- | --- | --- |
| Cycles delivered, median (IQR) | 4 (IQR 3–6) | 4 (IQR 3–6) |
| Completed ≥4 cycles, n (%) | 30 (62.5%) | 35 (66.0%) |
| Completed ≥6 cycles, n (%) | 18 (37.5%) | 14 (26.4%) |
| Discontinued <4 cycles, n (%) | 18 (37.5%) | 18 (34.0%) |
| Cisplatin-to-carboplatin switch, n (%) | 8 (16.7%) | — |

Panel B. Reasons for early discontinuation (<4 cycles) among patients who stopped early.

| Reason | RNU+GC (n=18) | RNU+ADC+ICI (n=18) |
| --- | --- | --- |
| Logistics/transfer | 2 (11.1%) | 6 (33.3%) |
| Patient decision/financial | 11 (61.1%) | 12 (66.7%) |
| Recurrence/progression | 3 (16.7%) | 0 (0.0%) |
| Toxicity/intolerance | 2 (11.1%) | 0 (0.0%) |

*Footnote:*

ADC+ICI, disitamab vedotin plus PD-1 inhibitor; GC, gemcitabine/cisplatin; IQR, interquartile range; RNU, radical nephroureterectomy.

**Supplementary Table 8.** Selected exploratory HER2 IHC category–stratified overlap-weighted Cox estimates in Cohort B

| Endpoint | HER2 IHC category | HR | 95% CI | Nominal P value | FDR-adjusted P value | Interpretation |
| --- | --- | --- | --- | --- | --- | --- |
| Conventional DFS | IHC 2+/3+ | 0.16 | 0.04–0.58 | 0.006 | 0.026 | Exploratory favourable signal |
| Non-intravesical PFS | IHC 2+/3+ | 0.12 | 0.03-0.50 | 0.004 | 0.026 | Exploratory favourable signal |
| OS | IHC 2+/3+ | 0.22 | 0.05-0.90 | 0.035 | 0.098 | Not significant after FDR adjustment |
| IVRFS | IHC 2+/3+ | 0.11 | 0.01-0.84 | 0.033 | 0.098 | Not significant after FDR adjustment |
| OS | IHC 0 | 0.94 | 0.05-17.62 | 0.969 | 0.969 |  |
| Conventional DFS | IHC 0 | 0.85 | 0.09-7.66 | 0.886 | 0.966 |  |
| Conventional DFS | IHC 1+ | 2.03 | 0.12-33.72 | 0.619 | 0.964 |  |
| Non-intravesical PFS | IHC 0 | 0.85 | 0.07-9.48 | 0.897 | 0.966 |  |
| Non-intravesical PFS | IHC 1+ | 1.49 | 0.08-29.20 | 0.790 | 0.966 |  |
| IVRFS | IHC 1+ | 1.50 | 0.08-29.20 | 0.790 | 0.965 |  |
| MFS | IHC 0 | 2.53 | 0.17-36.93 | 0.498 | 0.872 |  |

*Footnote:*

These subgroup analyses were exploratory. Formal treatment-by-HER2 interaction testing was not considered reliable for several endpoints because of sparse event counts within HER2 strata. Only estimable and interpretable subgroup estimates are shown. Estimates affected by sparse events, separation, or extreme instability were not reported.

**Supplementary Table 9.** Additional sensitivity analyses in Cohort B

Panel A. Sensitivity analysis by PD-1 inhibitor type within the ADC+ICI group

| Endpoint | Comparison | HR | 95% CI | Nominal P value | FDR-adjusted P value |
| --- | --- | --- | --- | --- | --- |
| OS | Tislelizumab vs toripalimab | 0.30 | 0.03-2.74 | 0.286 | 0.429 |
| Conventional DFS | Tislelizumab vs toripalimab | 0.26 | 0.06-1.19 | 0.083 | 0.166 |
| Non-intravesical PFS | Tislelizumab vs toripalimab | 0.14 | 0.02-1.21 | 0.074 | 0.166 |
| CSS | Tislelizumab vs toripalimab | 0.49 | 0.05-4.89 | 0.544 | 0.653 |
| MFS | Tislelizumab vs toripalimab | 0.73 | 0.11-5.03 | 0.751 | 0.751 |
| IVRFS | Tislelizumab vs toripalimab | - | - | - | - |

Panel B. Sensitivity analysis excluding GC patients who switched from cisplatin to carboplatin

| Endpoint | HR | 95% CI | Nominal P value | FDR-adjusted P value |
| --- | --- | --- | --- | --- |
| OS | 1.29 | 0.29-5.80 | 0.738 | 0.858 |
| Conventional DFS | 0.74 | 0.31-1.73 | 0.484 | 0.860 |
| Non-intravesical PFS | 0.51 | 0.20-1.35 | 0.176 | 0.527 |
| CSS | 1.52 | 0.25-9.21 | 0.648 | 0.858 |
| IVRFS | 0.27 | 0.05-1.43 | 0.123 | 0.527 |
| MFS | 0.89 | 0.23-3.35 | 0.858 | 0.858 |

*Footnote:*

Panel A was restricted to patients in the ADC+ICI group and compared tislelizumab with toripalimab. Panel B repeated the Cohort B comparative analysis after excluding GC patients who switched from cisplatin to carboplatin. Estimates with sparse events should be interpreted cautiously.

**Supplementary Fig. 1.** Study flow diagram.


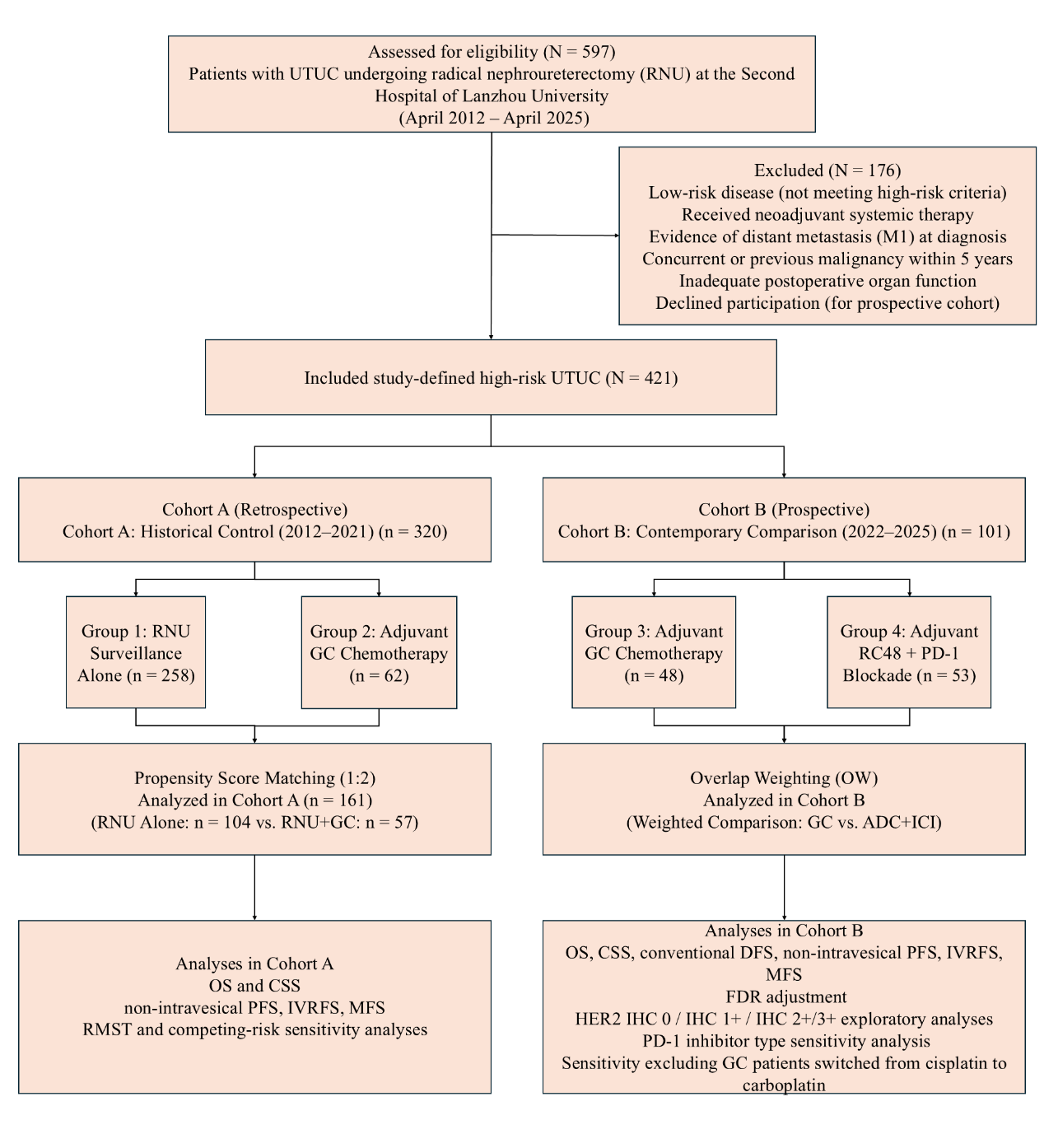


Supplementary Figure 1. Study flow diagram. A total of 597 patients with UTUC who underwent radical nephroureterectomy at Lanzhou University Second Hospital between April 2012 and April 2025 were assessed for eligibility. After exclusion, 421 patients with study-defined high-risk UTUC were included. Cohort A was a retrospective reference cohort comparing RNU alone with adjuvant GC after propensity score matching. Cohort B was a prospectively followed contemporary cohort comparing adjuvant GC with ADC+ICI using overlap weighting. In Cohort B, conventional DFS and non-intravesical PFS were reported separately, and HER2 was evaluated using three IHC categories: IHC 0, IHC 1+, and IHC 2+/3+.

ADC+ICI, disitamab vedotin plus PD-1 inhibitor; CSS, cancer-specific survival; DFS, disease-free survival; FDR, false discovery rate; GC, gemcitabine/cisplatin; IHC, immunohistochemistry; IVRFS, intravesical recurrence-free survival; MFS, metastasis-free survival; OS, overall survival; PFS, progression-free survival; PSM, propensity score matching; RNU, radical nephroureterectomy; UTUC, upper tract urothelial carcinoma.

**Supplementary Fig. 2.** Covariate balance before and after propensity score methods in Cohorts A and B.


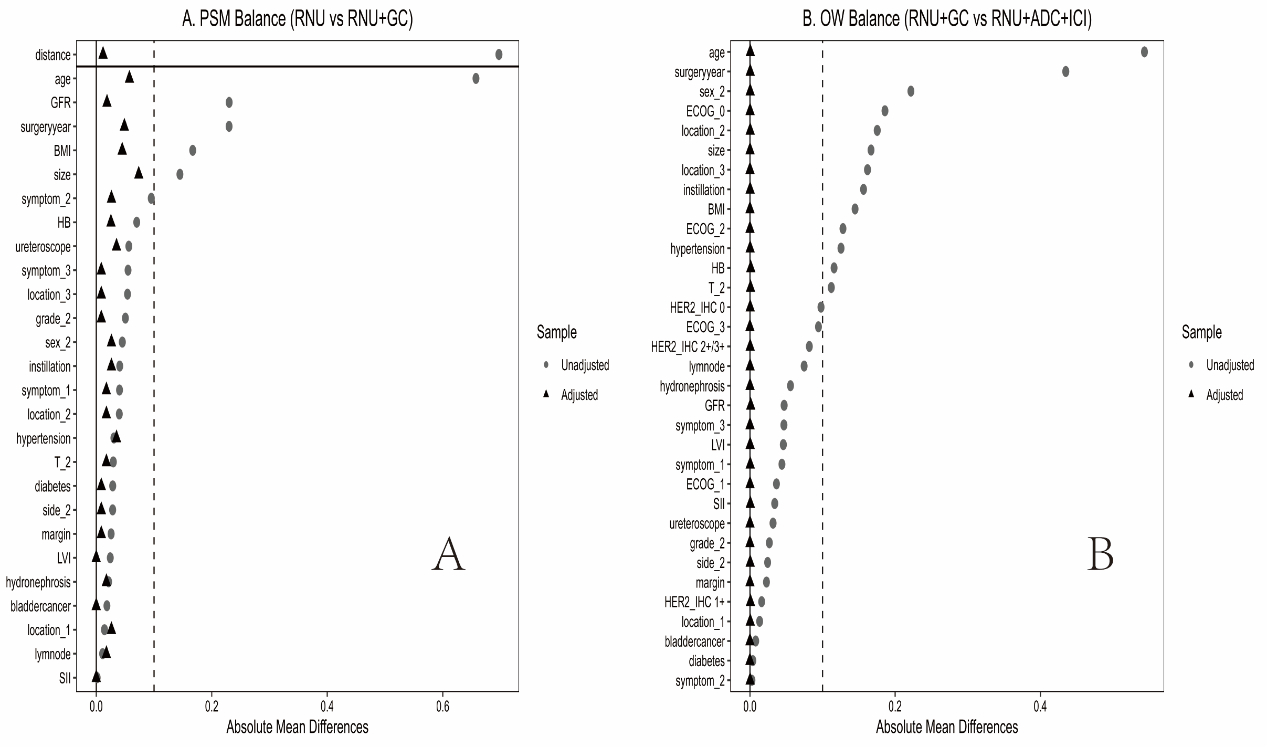


Supplementary Figure 2. Covariate balance before and after propensity score methods in Cohorts A and B.

(A) Standardized mean differences for baseline covariates before and after 1:2 propensity score matching in Cohort A, comparing RNU alone with RNU plus adjuvant GC.

(B) Standardized mean differences for baseline covariates before and after overlap weighting in Cohort B, comparing adjuvant GC with ADC+ICI. The updated Cohort B overlap-weighting model incorporated ECOG performance status, HER2 IHC category, treatment year, renal function, and other measured baseline covariates. Partial cisplatin-ineligibility based on eGFR/ECOG was additionally displayed as a balance descriptor. The dashed vertical line indicates an SMD of 0.10.

ADC+ICI, disitamab vedotin plus PD-1 inhibitor; eGFR, estimated glomerular filtration rate; GC, gemcitabine/cisplatin; IHC, immunohistochemistry; OW, overlap weighting; PSM, propensity score matching; RNU, radical nephroureterectomy; SMD, standardized mean difference.

**Supplementary Fig. 3.** Non-intravesical progression-free, intravesical recurrence-free, and metastasis-free survival in Cohort A before and after propensity score matching.


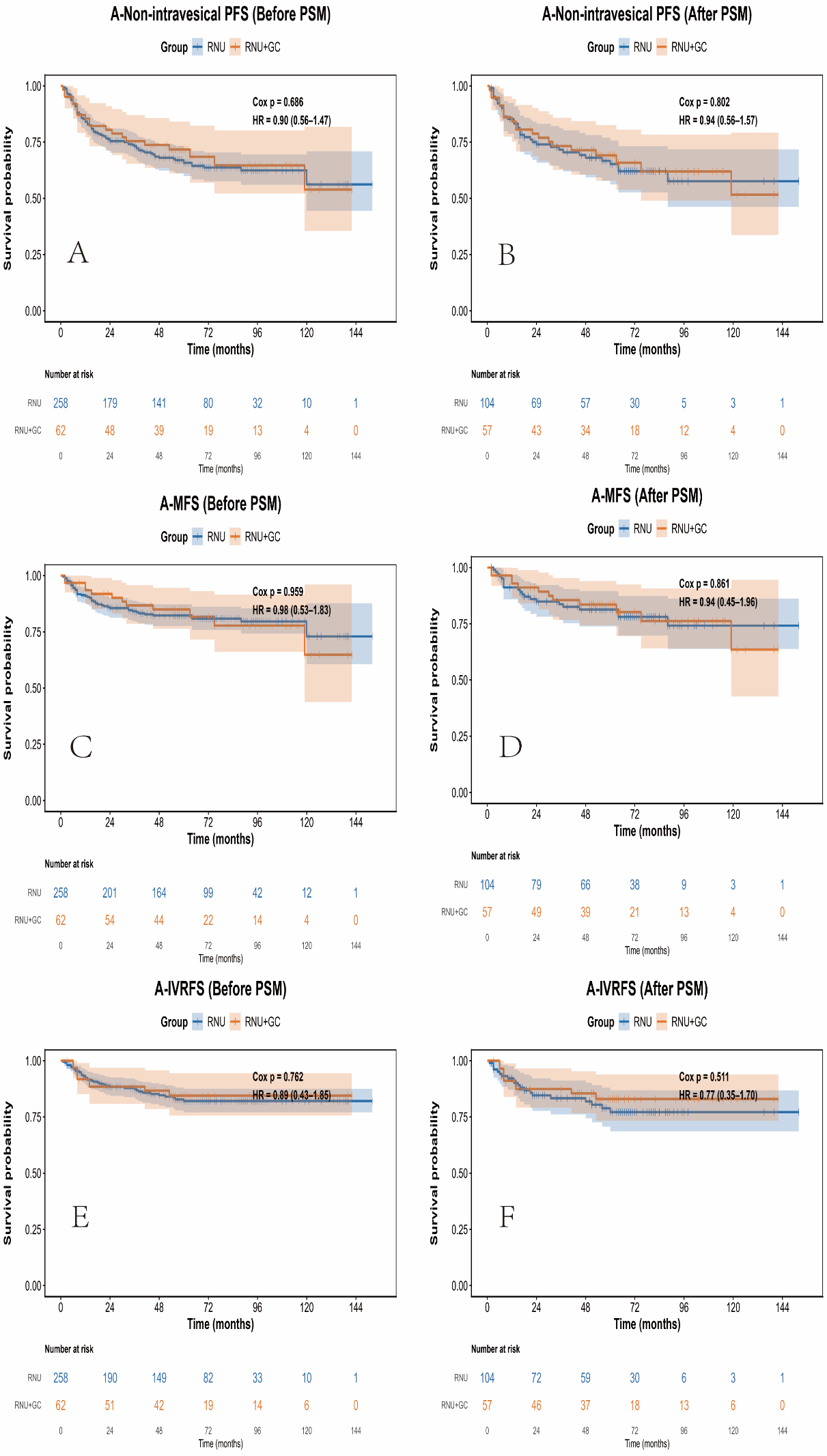


Supplementary Figure 3. Non-intravesical progression-free, intravesical recurrence-free, and metastasis-free survival in Cohort A before and after propensity score matching.

(A) Kaplan–Meier curves for non-intravesical PFS comparing RNU alone with RNU plus adjuvant GC before propensity score matching.

(B) Kaplan–Meier curves for non-intravesical PFS comparing RNU alone with RNU plus adjuvant GC after propensity score matching.

(C) Kaplan–Meier curves for IVRFS before propensity score matching.

(D) Kaplan–Meier curves for IVRFS after propensity score matching.

(E) Kaplan–Meier curves for MFS before propensity score matching.

(F) Kaplan–Meier curves for MFS after propensity score matching.

GC, gemcitabine/cisplatin; IVRFS, intravesical recurrence-free survival; MFS, metastasis-free survival; non-intravesical PFS, non-intravesical progression-free survival; PSM, propensity score matching; RNU, radical nephroureterectomy.

**Supplementary Fig. 4.** Exploratory three-month landmark analyses in Cohort A.


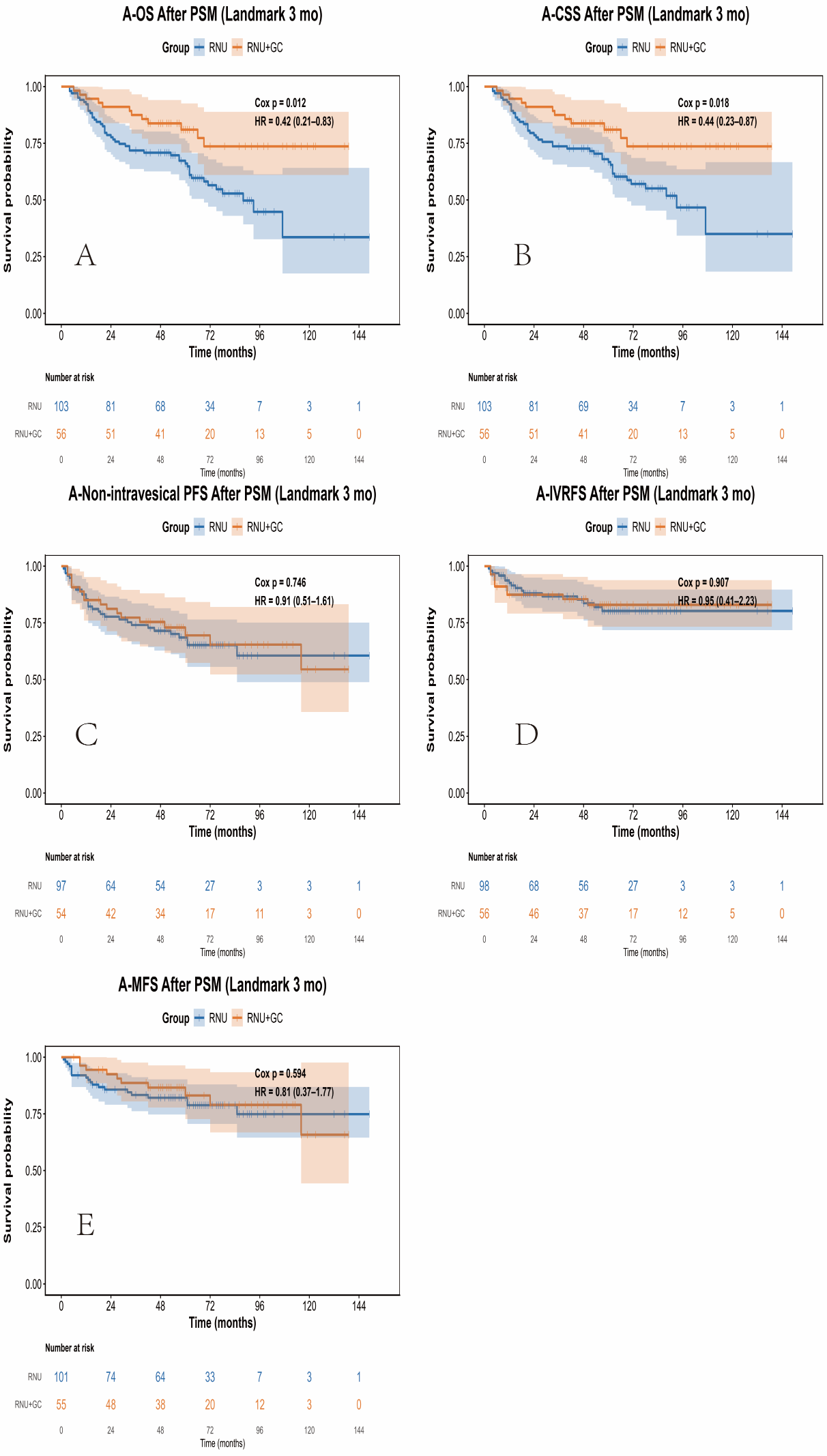


Supplementary Figure 4. Exploratory three-month landmark analyses in Cohort A.

(A) Overall survival.

(B) Cancer-specific survival.

(C) Non-intravesical progression-free survival.

(D) Intravesical recurrence-free survival.

(E) Metastasis-free survival.

Each analysis included only patients who were alive and event-free 3 months after RNU. These analyses were exploratory and were not used as the primary evidence of treatment effect.

CSS, cancer-specific survival; GC, gemcitabine/cisplatin; IVRFS, intravesical recurrence-free survival; MFS, metastasis-free survival; non-intravesical PFS, non-intravesical progression-free survival; OS, overall survival; RNU, radical nephroureterectomy.

**Supplementary Fig. 5.** Supplementary survival outcomes in Cohort B before and after overlap weighting.


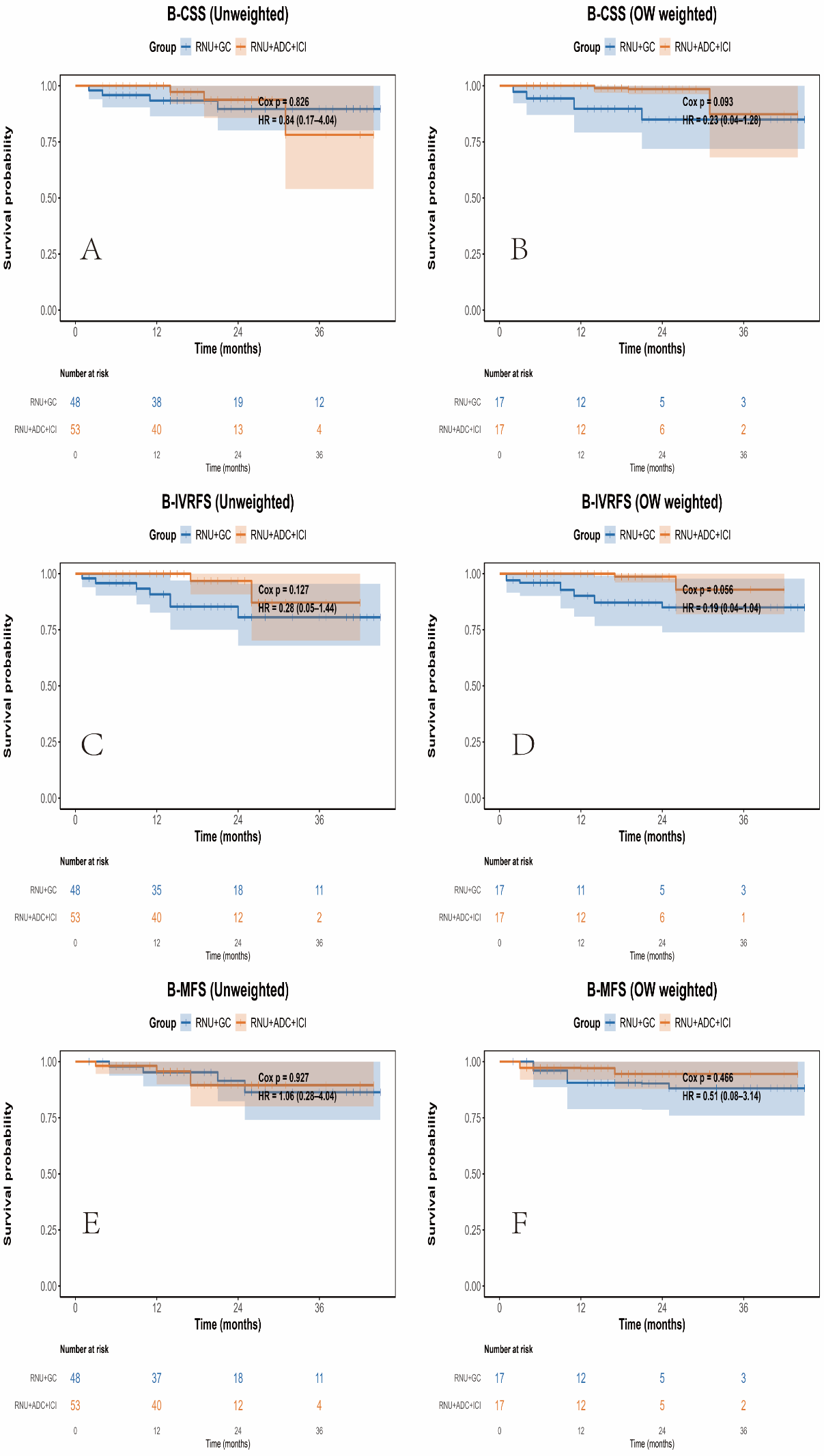


Supplementary Figure 5. Supplementary survival outcomes in Cohort B before and after overlap weighting.

(A) Unweighted Kaplan–Meier curves for CSS comparing adjuvant GC with ADC+ICI.

(B) Overlap-weighted Kaplan–Meier curves for CSS.

(C) Unweighted Kaplan–Meier curves for IVRFS.

(D) Overlap-weighted Kaplan–Meier curves for IVRFS.

(E) Unweighted Kaplan–Meier curves for MFS.

(F) Overlap-weighted Kaplan–Meier curves for MFS.

These analyses were supplementary and should be interpreted in the context of the limited number of events and effective sample size in Cohort B.

ADC+ICI, disitamab vedotin plus PD-1 inhibitor; CSS, cancer-specific survival; GC, gemcitabine/cisplatin; IVRFS, intravesical recurrence-free survival; MFS, metastasis-free survival; OW, overlap weighting.
